# Supplementary material for: Patient organisations’ views, motivations and experiences on patient involvement in cancer research: a pilot study in Portugal
Source: BMJ Open. 2024 Jan 24;14(1):e077444. doi: 10.1136/bmjopen-2023-077444 (PMC10824046; doi:10.1136/bmjopen-2023-077444)
Supplement: Supplementary data [file bmjopen-2023-077444supp001.pdf]

NOVA SCHOOL OF BUSINESS & ECONOMICS

English (United Kingdom)

Introduction

Dear Sir/Madam,

This study is part of the research project "**Patient Involvement in Research**", developed by a group of researchers and patient advocates, coordinated by Constança Roquette from the Nova School of Business and Economics, NOVA University of Lisbon. In the context of the Portuguese Presidency of the EU Council, and in response to the challenge posed by the *Europe: Unite against Cancer* declaration, this project aims to contribute to the understanding of **how patients and the organizations that represent them have been involved in research projects in the area of cancer, in Portugal.**

Therefore, a **questionnaire was developed for representatives of cancer patient organizations.** Through the responses obtained, it will be possible to analyze how organizations participate and are involved in research, as well as their perceptions, motivations and aspirations in this area.

To do this, we ask you to complete this questionnaire with 41 questions and an aproximate time of **30 minutes.**

Your participation in this study is **voluntary** and the non-participation will not cause you to be penalised. You can drop out of the study at any time.

Moreover, **the data from each organization will be encrypted** so their identity will not be assessed during data analysis. The **data will be clustered for analysis, rather than assessing the performance of each organization separately.** Only the Principal Investigator will have access to the encryption key, with the sole purpose of monitoring the number of responses.

This study received the approval of the Ethics Committee of the NOVA School of Business and Economics and is in full compliance with the General Data Protection Regulation (GDPR).**In case of any doubt, you can contact us** via email constanca.roquette@novasbe.pt.

From the results obtained with this questionnaire, **a space for joint and constructive reflection on the different opportunities for the involvement of patients in research will be created.** These will also be disclosed at a **meeting, to be held by the end of the Portuguese Presidency of the EU Council, in the 2nd quarter of this year**, where it is the intention to identify good practices, as well as discuss points of improvement for a more effective investigation that is closer to the real needs of patients.

If you want to be informed of the results and wish to be present at this meeting, fill out the registration form here.

We are very grateful for your participation.

I agree to participate in this study and confirm that I was informed about its conditions and that I have no questions in regard to it.

- ○ Yes.
- ○ No.

I answer this questionnaire on behalf of the following cancer patient organization:
(This information will be encrypted so the organization's identity will not be assessed during data analysis. The data will be clustered for analysis, rather than assessing the performance of each organization separately.)

[ ]

Do you identify yourself as the organization's usual representative?

- ○ Yes.

○ No. What is your role within the organization?

Clinical Research

Patients' participation in a clinical study *can occur at different stages, from participating in the design of the protocol, being present at meetings with researchers, participating in its dissemination during the recruitment phase to patients, disseminating results, among many others.*

Indicate the number and type of clinical studies your organization has been / is involved in.

(If you have not been involved in any of the types of studies presented, please mark with a "0" in the corresponding options.)

**Basic or pre-clinical studies** (laboratory studies to assess the biology of the disease or potential therapies and risks for humans. For example, pharmacological, pharmacokinetic and toxicological studies).	0
**Observational studies** (clinical studies without intervention, in which the participant is monitored by observation and data collection. It may, for example, be a study on the quality of life of people living with a certain disease / condition, by filling in a questionnaire).	0
**Clinical trials** (clinical studies with intervention, which may be any investigation conducted in humans, aimed at discovering or verifying the effects of one or more experimental drugs in order to determine their safety or efficacy).	0
**Others.** Which?	0
Total	0

What were the reasons that previously led to the non-participation of your organization in a clinical study?

- ☐ The organization was never asked to participate in this type of study.
- ☐ The organization was never able to respond to previous invitations to participate in a clinical study. Why?
- ☐ The organization does not consider its participation in a clinical study to be relevant.
- ☐ Other. Which?

Has your organization ever participated in another type of health research? (epidemiological, public health studies, etc.)

- ○ Yes.
- ○ No.

Who had the initiative to involve your organization in the clinical study(ies)?

- ☐ The initiative came from the organization itself. *(through an associate or a Board member, for example.)*
- ☐ The initiative came from others. *(such as a research team, an individual investigator, or a patient, for example.)*
- ☐ Other. Which?

Did the initiative to develop the study ever come from the organization itself?

- Yes.
- No.

Has the organization ever participated in the development of clinical recommendations?

- Yes.
- No.

Considering the clinical studies in which your organization was / is involved, select the option that represents the highest degree of involvement of your organization in each of the research stages.

Stage 1: Setting Research Priorities

At this stage, the organization:

	Did not participate or receive information (Level 0)	Has received information about this stage (Level 1)	Participated in information collection (Level 2)	Participated in the discussion of key points of the study (Level 3)	Participated in the discussion and actively decided together with the other partners (Level 4)	Participated as a full member of the research team, with equal decision-making power (Level 5)
Identification of the real needs of patients.	○	○	○	○	○	○

Stage 2: Research Design and Planning

At this stage, the organization:

	Did not participate or receive information (Level 0)	Has received information about this stage (Level 1)	Participated in information collection (Level 2)	Participated in the discussion of key points of the study (Level 3)	Participated in the discussion and actively decided together with the other partners (Level 4)	Participated as a full member of the research team, with equal decision-making power (Level 5)

	Did not participate or receive information (Level 0)	Has received information about this stage (Level 1)	Participated in information collection (Level 2)	Participated in the discussion of key points of the study (Level 3)	Participated in the discussion and actively decided together with the other partners (Level 4)	Participated as a full member of the research team, with equal decision-making power (Level 5)
**Study's synopsis design:** design and target population definition.	◯	◯	◯	◯	◯	◯
**Protocol design:** endpoints, inclusion and exclusion criteria, quality of life measures and patient-reported outcomes, crossover, ethical and data protection issues, recruitment and dissemination plan, retention measures, risk-benefit balance, etc.	◯	◯	◯	◯	◯	◯
**Consideration of practical aspects:** contractual issues, travel expenses and support for family members.	◯	◯	◯	◯	◯	◯
**Fundraising** for the research project.	◯	◯	◯	◯	◯	◯
**Patient Information sheet about the project:** content, visual design, readability, language and dissemination.	◯	◯	◯	◯	◯	◯
**Informed Consent:** content, visual design, readability and language.	◯	◯	◯	◯	◯	◯
**Ethical review** of the research project.	◯	◯	◯	◯	◯	◯

Stage 3: Research Conduct and Operations

At this stage, the organization:

	Did not participate or receive information (Level 0)	Has received information about this stage (Level 1)	Participated in information collection (Level 2)	Participated in the discussion of key points of the study (Level 3)	Participated in the discussion and actively decided together with the other partners (Level 4)	Participated as a full member of the research team, with equal decision-making power (Level 5)
**Investigators meeting to monitor the project:** representation of patients' perspectives on the study, recruitment, challenges, opportunities, which can trigger amendments.	◯	◯	◯	◯	◯	◯
**Trial Steering Committee:** protocol follow up, improvements in patient access and adherence to the study.	◯	◯	◯	◯	◯	◯
**Data and Safety Monitoring Committee:** risk / benefit assessment, drop-out issues and amendments to the project.	◯	◯	◯	◯	◯	◯

	Did not participate or receive information (Level 0)	Has received information about this stage (Level 1)	Participated in information collection (Level 2)	Participated in the discussion of key points of the study (Level 3)	Participated in the discussion and actively decided together with the other partners (Level 4)	Participated as a full member of the research team, with equal decision-making power (Level 5)
**Information to participants:** communication of protocol amendments and new safety information.	○	○	○	○	○	○
**Study reporting:** summary of interim results and dissemination in patient community.	○	○	○	○	○	○

Stage 4: Dissemination, Communication, Post-approval

At this stage, the organization:

	Did not participate or receive information (Level 0)	Has received information about this stage (Level 1)	Participated in information collection (Level 2)	Participated in the discussion of key points of the study (Level 3)	Participated in the discussion and actively decided together with the other partners (Level 4)	Participated as a full member of the research team, with equal decision-making power (Level 5)
**Health Technology Assessment:** assessment of value, patient-relevant outcomes and patient priorities.	○	○	○	○	○	○
**Regulatory affairs:** preparation of lay summaries of results, information leaflets and updated safety communications.	○	○	○	○	○	○
**Post-study communication:** contribution to publications, dissemination of research results to patient community / healthcare professionals.	○	○	○	○	○	○

What is your degree of agreement with the following statements regarding the overall participation of the organization in the clinical study(ies) in which it was / is involved?

	1. Strongly disagree.	2. Disagree.	3. Agree.	4. Strongly agree.	Rather not answer.
My organization's participation was valued by the research team in the course of the study.	○	○	○	○	○

	1. Strongly disagree.	2. Disagree.	3. Agree.	4. Strongly agree.	Rather not answer.
The contribution made by my organization was integrated / incorporated into the study.	○	○	○	○	○
My organization actively participated in the study.	○	○	○	○	○
There was an effective dialogue between my organization and the research team throughout the study.	○	○	○	○	○
My organization was recognized as a partner of equal relevance / weight in the study.	○	○	○	○	○
After participating in the study, the results were shared with my organization before being publicly disclosed.	○	○	○	○	○

Select the expressions that best represent the overall impact of your organization's participation in the clinical study(ies) in which it has been / is involved. (Select up to 3 expressions.)

- ☐ Increase in the relevance of the research.
- ☐ Increase in patient participation rates.
- ☐ Increase in the quality of the generated knowledged.
- ☐ Help in the better understanding of informed consent by the patients involved.
- ☐ Reduce in time for research completion.
- ☐ Reduce in financial costs of research.
- ☐ Minimize research participants risk.
- ☐ Others. Which?
- ☐ The impact of participation in the studies in which the organization was / is involved is unknown.

What main motivations do you identify in your organization to participate in clinical studies focused on the disease(s) to which it is dedicated? (Select up to 5 motivations.)

- [ ] Ensure that the study and its results address the real needs of patients.
- [ ] Gain research knowledge and skills.
- [ ] Increase the self-esteem and confidence of the patient(s) who represent the organization in the research process.
- [ ] Apply the experience and knowledge of patients about their condition.
- [ ] Promote the acceptance of patients as partners in a clinical study and increase the sense of co-creation of research.
- [ ] Increase access to finance that supports topics that would not otherwise be considered on the research agenda.
- [ ] Increase understanding of the nature and purpose of a clinical study.
- [ ] Improve understanding between patients and researchers.
- [ ] Develop health care and therapies that are more representative of patients' real needs.
- [ ] Promote data and information exchange between users and industry on the real use and management of a new drug / therapy (phase IV, pharmacovigilance).
- [ ] Others. Which?
- [ ] None of the above.

What is the interest of your organization in participating in the different stages of research* of the disease(s) to which it is dedicated?

	1. Not interested.	2. Somewhat interested.	3. Interested.	4. Very interested.	Rather not answer.
**1. Setting Research Priorities**					
Identification of the real needs of patients.	○	○	○	○	○
**2. Research Design and Planning**					
**Study's synopsis design:** design and target population definition.	○	○	○	○	○
**Protocol design:** endpoints, inclusion and exclusion criteria, quality of life measures and patient-reported outcomes, crossover, ethical and data protection issues, recruitment and dissemination plan, retention measures, risk-benefit balance, etc.	○	○	○	○	○

	1. Not interested.	2. Somewhat interested.	3. Interested.	4. Very interested.	Rather not answer.
**Consideration of practical aspects:** contractual issues, travel expenses and support for family members.	○	○	○	○	○
**Fundraising** for the research project.	○	○	○	○	○
**Patient Information sheet about the project:** content, visual design, readability, language and dissemination.	○	○	○	○	○
**Informed Consent:** content, visual design, readability and language.	○	○	○	○	○
**Ethical review** of the research project.	○	○	○	○	○
**3. Research Conduct and Operations**					
**Investigators meeting to monitor the project:** representation of patients' perspectives on the study, recruitment, challenges, opportunities, which can trigger amendments.	○	○	○	○	○
**Trial Steering Committee:** protocol follow up, improvements in patient access and adherence to the study.	○	○	○	○	○
**Data and Safety Monitoring Committee:** risk / benefit assessment, drop-out issues and amendments to the project.	○	○	○	○	○
**Information to participants:** communication of protocol amendments and new safety information.	○	○	○	○	○
**Study reporting:** summary of interim results and dissemination in patient community.	○	○	○	○	○
**4. Dissemination, Communication, Post-approval**					
**Health Technology Assessment:** assessment of value, patient-relevant outcomes and patient priorities.	○	○	○	○	○
**Regulatory affairs:** preparation of lay summaries of results, information leaflets and updated safety communications.	○	○	○	○	○
**Post-study communication:** contribution to publications, dissemination of research results to patient community / healthcare professionals.	○	○	○	○	○

What do you consider to be the influence of your organization in the different stages of research* of the disease(s) to which it is dedicated?

	1. Not influential.	2. Somewhat influential.	3. Influential.	4. Very influential.	Rather not answer.

	1. Not influential.	2. Somewhat influential.	3. Influential.	4. Very influential.	Rather not answer.
**1. Setting Research Priorities**					
Identification of the real needs of patients.	○	○	○	○	○
**2. Research Design and Planning**					
**Study's synopsis design:** design and target population definition.	○	○	○	○	○
**Protocol design:** endpoints, inclusion and exclusion criteria, quality of life measures and patient-reported outcomes, crossover, ethical and data protection issues, recruitment and dissemination plan, retention measures, risk-benefit balance, etc.	○	○	○	○	○
**Consideration of practical aspects:** contractual issues, travel expenses and support for family members.	○	○	○	○	○
**Fundraising** for the research project.	○	○	○	○	○
**Patient Information sheet about the project:** content, visual design, readability, language and dissemination.	○	○	○	○	○
**Informed Consent:** content, visual design, readability and language.	○	○	○	○	○
**Ethical review** of the research project.	○	○	○	○	○
**3. Research Conduct and Operations**					
**Investigators meeting to monitor the project:** representation of patients' perspectives on the study, recruitment, challenges, opportunities, which can trigger amendments.	○	○	○	○	○
**Trial Steering Committee:** protocol follow up, improvements in patient access and adherence to the study.	○	○	○	○	○
**Data and Safety Monitoring Committee:** risk / benefit assessment, drop-out issues and amendments to the project.	○	○	○	○	○
**Information to participants:** communication of protocol amendments and new safety information.	○	○	○	○	○
**Study reporting:** summary of interim results and dissemination in patient community.	○	○	○	○	○
**4. Dissemination, Communication, Post-approval**					
**Health Technology Assessment:** assessment of value, patient-relevant outcomes and patient priorities.	○	○	○	○	○
**Regulatory affairs:** preparation of lay summaries of results, information leaflets and updated safety communications.	○	○	○	○	○

	1. Not influential.	2. Somewhat influential.	3. Influential.	4. Very influential.	Rather not answer.
**Post-study communication:** contribution to publications, dissemination of research results to patient community / healthcare professionals.	○	○	○	○	○

What is your perception of the influence that the following factors may have on the involvement of patient organizations in clinical studies?

	1. Not influential.	2. Somewhat influential.	3. Influential.	4. Very influential.
Participation in national and international collaborative networks.	○	○	○	○
Participation in medical and scientific events.	○	○	○	○
Visibility of the organization among the different stakeholders in the area of the disease on which it focuses.	○	○	○	○
Search for current and evidence-based information.	○	○	○	○
Existence of a collaborative culture / experience exchange between organizations and public institutions.	○	○	○	○
Existence of bureaucratic requirements / administrative procedures for the health system.	○	○	○	○
Researchers / Clinitians believe that patients are not able to participate in research.	○	○	○	○
Organizations own belief that patients are not able to participate in research	○	○	○	○
Researchers' lack of knowledge about how to involve patients in research.	○	○	○	○
Recognition of the importance of involving patients in research by the different stakeholders in the area of the disease.	○	○	○	○
Use of accessible and clear language for patients during research.	○	○	○	○
Interventional / participatory profile of the organization.	○	○	○	○
Knowledge of society in general on the subject of clinical research.	○	○	○	○
Knowledge of patients and organizations about the research process, as a whole, and about its different stages, in particular.	○	○	○	○

As initially mentioned, the aim of this study is to create a **space for joint and constructive reflection on the different opportunities for patients to be involved in research.**

Therefore, a **meeting will take place, by the end of the Portuguese Presidency of the Council of the EU, in the 2nd quarter of this year**, where it is the intention to identify good practices, as well as to discuss points of improvement for more effective research that is closer to the real needs of the patients.

In order to better prepare this meeting, we would like to ask you some questions.

How do you think your organization would like to be more involved in clinical research? (areas, projects or research phases, collaborations, etc.)

What clinical research topics would you like to discuss with other stakeholders in this area?

In what areas do you think that greater capacity is needed for patient organizations for better participation and more effective clinical research?

What would you like to say about your experience with bad practices in involving patients in research? What major problems can you identify?

Organization's Characteristics

This second part of the questionnaire aims to characterize your organization.

Organization Type

- ☐ Association.
- ☐ Foundation.
- ☐ Associative movement or other informal organization.
- ☐ Estatuto de Utilidade Pública. ****
- ☐ IPSS. ***
- ☐ NPO

Portuguese context..

Does the organization have a Scientific Advisory Board?

- ○ Yes.
- ○ No.

Do the organization's governing bodies include people with disease they represent?

	Yes	No
Board of Directors	○	○
Other governing bodies	○	○

Type of disease(s) to which the organization focuces on.

- ☐ Rare disease(s).
- ☐ Non rare disease(s).
- ☐ Single pathology.
- ☐ Multiple pathology.

Organization's foundation year.

Annual budget *(based on the average value for the last 3 years, 2020 may be excluded if it is not representative, due to the Covid-19 pandemic).*

- ○ Up to 100 thousand euros.
- ○ 100 thousand to 250 thousand euros.
- ○ 250 thousand to 500 thousand euros.
- ○ 500 thousand to 1 million euros.
- ○ 1 million euros or more.

Number of associate members.

- ○ Less than 100 associate members.
- ○ 100 to 499 associate members.
- ○ 500 to 2999 associate members.
- ○ 3000 or more associate members.

Number of regular employees (estimate).

	0	1-3	4-10	11-30	> 30
Number of volunteer employees.	○	○	○	○	○
Number of paid employees.	○	○	○	○	○

Activities that best represent the organization's work.

- ☐ Development of educational materials.
- ☐ Organization of support groups.
- ☐ Organization of events for the patient education and other audiences.
- ☐ Financing of events for healthcare professionals.
- ☐ Financial assistance to patients.
- ☐ Advocacy / activism at the political level.
- ☐ Participation in conferences.
- ☐ Publications.
- ☐ Training for entities that are external to the organization.
- ☐ Collaboration in research projects.

☐ Others. Which?

Preferred sources of consultation for building knowledge / thinking and the frequency with which you consult them.

	1. Never.	2. Rarely.	3. Often.	4. Always.	Rather not answer.
Websites of national government institutions.	○	○	○	○	○
Websites of international organizations (World Health Organization, European Center for Disease Prevention and Control, etc.).	○	○	○	○	○
Scientific publications.	○	○	○	○	○
News in newspapers or magazines.	○	○	○	○	○
TV News.	○	○	○	○	○
Free search on the Internet (Google, Youtube, Podcasts).	○	○	○	○	○
Radio.	○	○	○	○	○
Participation in conferences / workshops / seminars.	○	○	○	○	○
Universities through contact with scientists and researchers.	○	○	○	○	○
Private companies (pharmaceutical, food laboratories, etc.)	○	○	○	○	○
Friends and / or family.	○	○	○	○	○
Healthcare professionals.	○	○	○	○	○

Thank you very much for your time and availability to answer these questions.

We would like to know a little more about your organization and, for that, we have 8 more questions that take about 5 minutes to answer.

Are you willing to continue a little further?

- ○ Yes.
- ○ No.

Geographic scope.

- ☐ National.
- ☐ Regional.
- ☐ Local.
- ☐ International.

Main sources of funding *(select the organization's top three sources of funding).*

- ☐ Membership fees from associates.
- ☐ Individual donations / fund-raising campaign.
- ☐ Management of the organization's equity or own funds.
- ☐ Services provision.
- ☐ European / International financing.
- ☐ Financing of private organizations (pharmaceutical industry, private companies, etc.)
- ☐ Public financing (Ministry of Health, Social Security, etc.)
- ☐ Personal income tax assignment.
- ☐ Others.

Associate members profile *(please also indicate the estimated percentage of members by type of situation that best characterizes them).*

Patients.	0 %
Relatives.	0 %

Healthcare professionals.	0	%
Others.	0	%
Total	0	%

Services available to associate members / community.

- ☐ Treatments.
- ☐ Internment.
- ☐ Clinical appointments.
- ☐ Nutrition consultations.
- ☐ Examinations / clinical analysis / screenings.
- ☐ Information about the disease.
- ☐ Psychological support.
- ☐ Assistance support.
- ☐ Economic and financial support.
- ☐ Information about legal rights.
- ☐ Residential support.
- ☐ Protocols for reimbursement and discounts (drugs, etc).
- ☐ Rental and availability of spaces.
- ☐ Services provided to the community (catering, laundry,….)
- ☐ Others. Which?

Communication channels with associate members and the community.

	Associate members	Community	Not used
Website / blog.	☐	☐	☐
Newsletter / bulletin / newspaper.	☐	☐	☐
Flyers / brochures.	☐	☐	☐
Email.	☐	☐	☐

	Associate members	Community	Not used
Telephone.	☐	☐	☐
Twitter.	☐	☐	☐
Facebook.	☐	☐	☐
Instagram.	☐	☐	☐
Linkedin.	☐	☐	☐
Youtube.	☐	☐	☐
Whatsapp.	☐	☐	☐

Does the organization have the following departments?

	Yes - Internal Department.	Yes - Outsourced.	No.
Marketing Department.	☐	☐	☐
Communications Department.	☐	☐	☐
Department of Public Relations.	☐	☐	☐

Does your organization participate in any National / European / International collaborative network?

	Yes.	No.
National	○	○
European / International	○	○

What collaborations are most relevant to the organization's day-to-day activities? (select until 5 collborations)

- ☐ Other patient organizations.
- ☐ National Directorate-General for Health
- ☐ Hospital Healthcare
- ☐ Private Sector / Companies / Pharmaceutical Laboratories
- ☐ Academia / Research Centers / Education Institutions

- ☐ Administrações Regionais de Saúde (ARS)
- ☐ Serviços Partilhados do Ministério da Saúde (SPMS)
- ☐ Academia / Centros de Investigação / Instituições de Educação.
- ☐ Sector Social / Fundações / Organizações sem fins lucrativos
- ☐ Sector Privado / Empresas / Laboratórios Farmacêuticos
- ☐ Cuidados de Saúde Primários.
- ☐ Cuidados de Saúde Hospitalares.
- ☐ Comissão de Ética para a Saúde (CES) externa.
- ☐ Instituto Nacional de Saúde Doutor Ricardo Jorge (INSA).
- ☐ Outra(s). Qual(ais)?

Portuguese context...

End

Thank you very much for your time and availability to answer this questionnaire!

As initially mentioned, the aim of this project is to create a **space for joint and constructive reflection on the different opportunities for patients to be involved in research.** The results of this questionnaire will be disclosed at a **meeting, to be held by the end of the Presidency, in the 2nd quarter of this year**, where it is the intention to identify good practices, as well as to discuss points of improvement for a more effective investigation that is closer to the real needs of patients.

If you would like to be informed of the results and wish to be present at this meeting, fill out the registration form here.

Powered by Qualtrics
