# Supplementary material for: Patient organisations’ views, motivations and experiences on patient involvement in cancer research: a pilot study in Portugal
Source: BMJ Open. 2024 Jan 24;14(1):e077444. doi: 10.1136/bmjopen-2023-077444 (PMC10824046; doi:10.1136/bmjopen-2023-077444)
Supplement: Supplementary data [file bmjopen-2023-077444supp002.pdf]

NOVA SCHOOL OF BUSINESS & ECONOMICS

Português

Introdução

Exmo.(a) Sr.(a),

Este estudo insere-se no projeto de investigação “**Envolvimento de Doentes em Investigação**”, desenvolvido por um grupo de investigadores e representantes das pessoas que vivem com doença, coordenado por Constança Roquette da NOVA School of Business and Economics. No contexto da Presidência Portuguesa do Conselho da UE, e em resposta ao desafio colocado pela declaração *Europe: Unite against Cancer*, este projeto pretende contribuir para o entendimento da forma **como doentes e as organizações que os representam têm vindo a ser envolvidos em projetos de investigação clínica, na área do cancro, em Portugal.**

Deste modo, foi desenvolvido um **questionário dirigido a representantes de organizações de doentes oncológicos.** Através das respostas obtidas, será possível analisar como as organizações participam e são envolvidas em investigação, bem como as suas percepções, motivações e aspirações nesta área.

Para tal, pedimos-lhe que preencha este questionário, com **41 perguntas e tempo aproximado de 30 minutos.**

A sua participação neste estudo é **voluntária** e a não participação não lhe trará qualquer prejuízo. Poderá desistir de participar neste estudo a qualquer momento.

A informação recolhida destina-se unicamente a fins de investigação e será somente tratada pelos investigadores. **Os dados serão tratados de forma codificada**, de modo a que a identidade da organização não seja associada às suas respostas. Os **dados serão tratados de forma agrupada, sem nunca ser analisada cada organização, em particular**. Apenas terá acesso à chave de codificação a Investigadora Principal do estudo, com o intuito único de

monitorização do número de respostas.

Este estudo mereceu o parecer favorável da Comissão de Ética da NOVA School of Business and Economics e está em total conformidade com o Regulamento Geral da Proteção de Dados (RGPD). **Em caso de dúvida poderá contactar-nos** através do e-mail constanca.roquette@novasbe.pt.

A partir dos resultados obtidos com este questionário, será criado um **espaço de reflexão conjunta e construtiva sobre as diferentes oportunidades de envolvimento de doentes em investigação.** Estes serão, também, divulgados num **encontro, a realizar até ao final da Presidência Portuguesa do Conselho da UE, no 2º trimestre deste ano**, onde se pretenderá identificar boas práticas, bem como discutir pontos de melhoria para uma investigação mais eficaz e mais próxima das necessidades reais dos doentes.

Caso pretenda ser informado dos resultados e deseje estar presente neste encontro, preencha o formulário de inscrição aqui.

Agradecemos a sua participação.

Aceito participar neste estudo, confirmando que fui esclarecido(a) sobre as condições do mesmo e que não tenho dúvidas.

- ○ Sim.
- ○ Não.

Respondo a este questionário em representação da seguinte organização de doentes oncológicos:
(Esta informação será codificada, de modo a não ser associada às suas respostas. Os dados serão tratados de forma agrupada, sem nunca ser analisada cada organização, em particular.)

Identifica-se como o representante habitual da organização?

- ○ Sim.

○ Não. Qual a sua função dentro da organização?

Investigação Clínica

*A **participação de doentes num estudo clínico** pode ocorrer em diferentes etapas do mesmo, desde participar na concepção do protocolo, estar presente em reuniões com investigadores, participar na sua divulgação durante a fase de recrutamento a doentes, disseminar resultados, entre muitas outras.*

Indique o número e tipo de estudos clínicos em que a sua organização esteve/está envolvida.
(Em caso de não ter estado envolvida em algum, ou nenhum, dos tipos de estudos apresentados, assinale, por favor, com um "0" nas opções correspondentes.)

**Estudos básicos ou pré-clínicos** *(estudos laboratoriais para avaliação da biologia da doença ou potenciais terapias e riscos para o ser humano. Por exemplo, estudos farmacológicos, farmacocinéticos e toxicológicos).*	0
**Estudos observacionais** *(estudos clínicos sem intervenção, em que é feito o acompanhamento do participante por observação e recolha de dados. Poderá, por exemplo, ser um estudo sobre a qualidade de vida das pessoas que vivem com determinada doença/condição, feito através do preenchimento de um questionário).*	0
**Ensaios clínicos** *(estudos clínicos com intervenção, podendo ser qualquer investigação conduzida no ser humano, destinada a descobrir ou a verificar os efeitos de um ou mais medicamentos experimentais a fim de apurar a respetiva segurança ou eficácia).*	0
**Outros.** Quais?	0
Total	0

Quais os motivos que levaram, anteriormente, à não participação da sua organização num estudo clínico?

- ☐ A organização nunca foi solicitada para participar neste tipo de estudo.
- ☐ A organização não teve possibilidade para dar resposta a anteriores convites para participar num estudo clínico. Porquê?
- ☐ A organização não considera relevante a sua participação num estudo clínico.
- ☐ Outra. Qual?

A sua organização alguma vez participou num outro tipo de investigação em saúde? (estudos epidemiológicos, de saúde pública, etc.)

- ○ Sim.
- ○ Não.

De quem partiu a iniciativa de envolver a sua organização no(s) estudo(s) clínico(s)?

- ☐ A iniciativa partiu da própria organização.
(através de um associado ou de um membro Direcção, por exemplo.)
- ☐ A iniciativa partiu de outros.
(como uma equipa de investigação, um investigador a título individual ou um doente, por exemplo.)
- ☐ Outro. Qual?

Em alguma das vezes, a iniciativa de desenvolver o estudo partiu da própria organização?

- Sim.
- Não.

A organização participou alguma vez na elaboração de recomendações clínicas?

- Sim.
- Não.

Considerando os estudos clínicos em que a sua organização esteve/está envolvida, assinale a opção que represente o maior grau de envolvimento da sua organização em cada uma das etapas de investigação.

Etapa 1: Definição das prioridades de investigação.

Nesta etapa, a organização:

	Não participou nem recebeu informações (Nível 0)	Recebeu informações sobre esta etapa (Nível 1)	Participou na recolha de informações (Nível 2)	Participou na discussão de pontos-chave (Nível 3)	Participou na discussão e decidiu ativamente, em conjunto com os outros parceiros (Nível 4)	Participou como membro *de facto* da equipa de investigação, com igual poder de decisão (Nível 5)
Identificação das necessidades reais dos doentes.	○	○	○	○	○	○

Etapa 2: Desenho e planeamento do projeto de investigação

Nesta etapa, a organização:

	Não participou nem recebeu informações (Nível 0)	Recebeu informações sobre esta etapa (Nível 1)	Participou na recolha de informações (Nível 2)	Participou na discussão de pontos-chave (Nível 3)	Participou na discussão e decidiu ativamente, em conjunto com os outros parceiros (Nível 4)	Participou como membro *de facto* da equipa de investigação, com igual poder de decisão (Nível 5)
**Elaboração da sinopse / sumário do estudo:** desenho e definição da população alvo.	○	○	○	○	○	○
**Elaboração do protocolo:** definição de parâmetros relevantes, critérios de inclusão e exclusão, medidas de qualidade de vida e resultados reportados pelos doentes (*patient-reported outcomes*), desenho do estudo, questões de ética e de proteção de dados, plano de recrutamento e disseminação do estudo, medidas de retenção, análise risco-benefício, etc.	○	○	○	○	○	○
**Consideração de aspectos práticos:** questões contratuais, despesas de viagem/deslocação e apoio a familiares.	○	○	○	○	○	○
**Angariação de fundos** para o projeto de investigação.	○	○	○	○	○	○
**Folha de Informação aos doentes sobre o projeto:** conteúdo, design visual, legibilidade, linguagem e disseminação.	○	○	○	○	○	○
**Consentimento Informado**: conteúdo, *design* visual, legibilidade e linguagem.	○	○	○	○	○	○
**Revisão ética** do projeto de investigação.	○	○	○	○	○	○

Etapa 3: Condução da investigação e processos associados

Nesta etapa, a organização:

	Não participou nem recebeu informações (Nível 0)	Recebeu informações sobre esta etapa (Nível 1)	Participou na recolha de informações (Nível 2)	Participou na discussão de pontos-chave (Nível 3)	Participou na discussão e decidiu ativamente, em conjunto com os outros parceiros (Nível 4)	Participou como membro *de facto* da equipa de investigação, com igual poder de decisão (Nível 5)
**Reunião de Investigadores para acompanhamento do projeto:** representação das perspetivas dos doentes quanto ao estudo, recrutamento, desafios, oportunidades, podendo originar emendas.	○	○	○	○	○	○

	Não participou nem recebeu informações (Nível 0)	Recebeu informações sobre esta etapa (Nível 1)	Participou na recolha de informações (Nível 2)	Participou na discussão de pontos-chave (Nível 3)	Participou na discussão e decidiu ativamente, em conjunto com os outros parceiros (Nível 4)	Participou como membro *de facto* da equipa de investigação, com igual poder de decisão (Nível 5)
**Comissão de gestão / monitorização do estudo:** seguimento *(follow up)* do protocolo, melhorias no acesso e adesão dos doentes ao estudo.	○	○	○	○	○	○
**Comissão de Monitorização da Segurança dos Dados**: avaliação de riscos/benefícios, motivos de desistências e emendas ao projeto.	○	○	○	○	○	○
**Informação aos participantes:** comunicação de emendas do projeto e novas informações de segurança.	○	○	○	○	○	○
**Relatório do estudo:** sumário de resultados provisórios, disseminação pela comunidade de doentes.	○	○	○	○	○	○

Etapa 4: Disseminação, Comunicação e Atividades Pós-Aprovação.

Nesta etapa, a organização:

	Não participou nem recebeu informações (Nível 0)	Recebeu informações sobre esta etapa (Nível 1)	Participou na recolha de informações (Nível 2)	Participou na discussão de pontos-chave (Nível 3)	Participou na discussão e decidiu ativamente, em conjunto com os outros parceiros (Nível 4)	Participou como membro *de facto* da equipa de investigação, com igual poder de decisão (Nível 5)
**Avaliação de Tecnologias de Saúde (*Health Technology Assessment*):** avaliação de valor, relevância de resultados e prioridades para os doentes.	○	○	○	○	○	○
**Assuntos regulamentares:** elaboração de resumos em linguagem leiga, folhetos informativos e comunicações de segurança atualizadas.	○	○	○	○	○	○
**Comunicação após estudo:** contribuição para publicações, disseminação de resultados do estudo na comunidade de doentes e/ou profissionais de saúde.	○	○	○	○	○	○

Qual o seu grau de concordância com as seguintes afirmações relativamente à globalidade da participação da organização no(s) estudo(s) clínico(s) em que esteve/está envolvida?

	1. Discordo fortemente.	2. Discordo.	3. Concordo.	4. Concordo fortemente.	Pr[illegible] resp[illegible]
A participação da minha organização foi valorizada pela equipa de investigação no decorrer do estudo.	○	○	○	○	
O contributo dado pela minha organização foi integrado/incorporado no estudo.	○	○	○	○	
A minha organização participou ativamente na investigação.	○	○	○	○	
Houve efetivo diálogo entre a minha organização e a equipa de investigadores ao longo do estudo.	○	○	○	○	
A minha organização foi reconhecida como parceira de igual relevância/peso no estudo.	○	○	○	○	
Após participação no estudo, os resultados foram partilhados com a minha organização antes de serem publicamente divulgados.	○	○	○	○	

Selecione as expressões que melhor representam o impacto global da participação da sua organização no(s) estudo(s) clínicos em que esteve/está envolvida. (Selecione até 3 expressões.)

- ☐ Aumento da relevância da investigação.
- ☐ Aumento da taxa de participação de doentes.
- ☐ Aumento da qualidade do conhecimento gerado.
- ☐ Ajuda na melhor compreensão do consentimento informado por parte dos doentes envolvidos.
- ☐ Diminuição do tempo necessário para completar o estudo.
- ☐ Diminuição dos custos do estudo.
- ☐ Minimização dos riscos de participação para os doentes.
- ☐ Outros. Quais?

- ☐ O impacto da participação nos estudos em que a organização esteve envolvida é desconhecido.

Que principais motivações identifica na sua organização para participar em estudos clínicos sobre a(s) doença(s) a que se dedica? (Selecione até 5 motivações.)

- ☐ Garantir que o estudo e os seus resultados abordam reais necessidades dos doentes.
- ☐ Ganhar conhecimento e competências de investigação.
- ☐ Aumentar a auto-estima e confiança do(s) doente(s) que representa(m) a organização no processo de investigação.
- ☐ Aplicar a experiência e conhecimento dos doentes sobre a sua condição.
- ☐ Promover a aceitação dos doentes como parceiros num estudo clínico e aumento de sentido de co-criação da investigação.
- ☐ Aumentar o acesso a financiamento que permita colocar na agenda de investigação tópicos que de outro modo não seriam considerados.
- ☐ Aumentar a compreensão da natureza e propósito de um estudo clínico.
- ☐ Melhorar o entendimento entre doentes e investigadores.
- ☐ Desenvolver cuidados de saúde e terapias que são mais representativas das reais necessidades dos doentes.
- ☐ Promover a troca de dados e informações entre utilizadores e indústria sobre a realidade do uso e gestão de um novo medicamento / terapia (fase IV, farmacovigilância).
- ☐ Outros. Quais?
- ☐ Nenhumas.

Qual o interesse da sua organização em participar nas diferentes etapas de investigação da(s) doença(s) a que se dedica?

	1. Nada interessada.	2. Pouco interessada.	3. Interessada.	4. Muito interessada.	Prefiro não responder.

	1. Nada interessada.	2. Pouco interessada.	3. Interessada.	4. Muito interessada.	Prefiro não responder.
**1. Definição das prioridades de investigação**					
Identificação das necessidades reais dos doentes.	○	○	○	○	○
**2. Desenho e planeamento do projeto de investigação**					
**Elaboração da sinopse / sumário do estudo:** desenho e definição da população alvo.	○	○	○	○	○
**Elaboração do protocolo:** definição de parâmetros relevantes, critérios de inclusão e exclusão, medidas de qualidade de vida e resultados reportados pelos doentes (*patient-reported outcomes*), desenho do estudo, questões de ética e de proteção de dados, plano de recrutamento e disseminação do estudo, medidas de retenção, análise risco-benefício, etc.	○	○	○	○	○
**Consideração de aspectos práticos:** questões contratuais, despesas de viagem/deslocação e apoio a familiares.	○	○	○	○	○
**Angariação de fundos** para o projeto de investigação.	○	○	○	○	○
**Folha de Informação aos doentes sobre o projeto:** conteúdo, design visual, legibilidade, linguagem e disseminação.	○	○	○	○	○
**Consentimento Informado**: conteúdo, *design* visual, legibilidade e linguagem.	○	○	○	○	○
**Revisão ética** do projeto de investigação.	○	○	○	○	○
**3. Condução da investigação e processos associados**					
**Reunião de Investigadores para acompanhamento do projeto:** representação das perspetivas dos doentes quanto ao estudo, recrutamento, desafios, oportunidades, podendo originar emendas.	○	○	○	○	○
**Comissão de gestão / monitorização do estudo:** seguimento *(follow up)* do protocolo, melhorias no acesso e adesão dos doentes ao estudo.	○	○	○	○	○
**Comissão de Monitorização da Segurança dos Dados**: avaliação de riscos/benefícios, motivos de desistências e emendas ao projeto.	○	○	○	○	○
**Informação aos participantes:** comunicação de emendas do projeto e novas informações de segurança.	○	○	○	○	○
**Relatório do estudo:** sumário de resultados provisórios, disseminação pela comunidade de doentes.	○	○	○	○	○

	1. Nada interessada.	2. Pouco interessada.	3. Interessada.	4. Muito interessada.	Prefiro não responder.
**4. Disseminação, Comunicação e Atividades Pós-Aprovação**					
**Avaliação de Tecnologias de Saúde (*Health Technology Assessment*)**: avaliação de valor, relevância de resultados e prioridades para os doentes.	O	O	O	O	O
**Assuntos regulamentares:** elaboração de resumos em linguagem leiga, folhetos informativos e comunicações de segurança atualizadas.	O	O	O	O	O
**Comunicação após estudo:** contribuição para publicações, disseminação de resultados do estudo na comunidade de doentes e/ou profissionais de saúde.	O	O	O	O	O

Qual considera ser a influência da sua organização nas diferentes etapas de investigação da(s) doença(s) a que se dedica?

	1. Nada influente.	2. Pouco influente.	3. Influente.	4. Muito influente.	Prefiro não responder.
**1. Definição das prioridades de investigação**					
Identificação das necessidades reais dos doentes.	O	O	O	O	O
**2. Desenho e planeamento do projeto de investigação**					
**Elaboração da sinopse / sumário do estudo:** desenho e definição da população alvo.	O	O	O	O	O
**Elaboração do protocolo:** definição de parâmetros relevantes, critérios de inclusão e exclusão, medidas de qualidade de vida e resultados reportados pelos doentes (*patient-reported outcomes*), desenho do estudo, questões de ética e de proteção de dados, plano de recrutamento e disseminação do estudo, medidas de retenção, análise risco-benefício, etc.	O	O	O	O	O
**Consideração de aspectos práticos:** questões contratuais, despesas de viagem/deslocação e apoio a familiares.	O	O	O	O	O
**Angariação de fundos** para o projeto de investigação.	O	O	O	O	O
**Folha de Informação aos doentes sobre o projeto:** conteúdo, design visual, legibilidade, linguagem e disseminação.	O	O	O	O	O

	1. Nada influente.	2. Pouco influente.	3. Influente.	4. Muito influente.	Prefiro não responder.
**Consentimento Informado**: conteúdo, *design* visual, legibilidade e linguagem.	O	O	O	O	O
**Revisão ética** do projeto de investigação.	O	O	O	O	O
**3. Condução da investigação e processos associados**					
**Reunião de Investigadores para acompanhamento do projeto:** representação das perspetivas dos doentes quanto ao estudo, recrutamento, desafios, oportunidades, podendo originar emendas.	O	O	O	O	O
**Comissão de gestão / monitorização do estudo:** seguimento *(follow up)* do protocolo, melhorias no acesso e adesão dos doentes ao estudo.	O	O	O	O	O
**Comissão de Monitorização da Segurança dos Dados**: avaliação de riscos/benefícios, motivos de desistências e emendas ao projeto.	O	O	O	O	O
**Informação aos participantes:** comunicação de emendas do projeto e novas informações de segurança.	O	O	O	O	O
**Relatório do estudo:** sumário de resultados provisórios, disseminação pela comunidade de doentes.	O	O	O	O	O
**4. Disseminação, Comunicação e Atividades Pós-Aprovação**					
**Avaliação de Tecnologias de Saúde (*Health Technology Assessment)*:** avaliação de valor, relevância de resultados e prioridades para os doentes.	O	O	O	O	O
**Assuntos regulamentares:** elaboração de resumos em linguagem leiga, folhetos informativos e comunicações de segurança atualizadas.	O	O	O	O	O
**Comunicação após estudo:** contribuição para publicações, disseminação de resultados do estudo na comunidade de doentes e/ou profissionais de saúde.	O	O	O	O	O

Qual a sua perceção sobre a influência, negativa ou positiva, que podem ter os seguintes fatores no envolvimento das organizações de doentes em estudos clínicos?

	1. Nada influente.	2. Pouco influente.	3. Influente.	4. Muito influente.	Prefiro não responder.
Participação em redes colaborativas nacionais e internacionais.	O	O	O	O	O
Participação em congressos e eventos da comunidade médica e científica.	O	O	O	O	O

	1. Nada influente.	2. Pouco influente.	3. Influente.	4. Muito influente.	Prefiro não responder.
Visibilidade da organização entre os diferentes atores da área da doença em que se foca.	O	O	O	O	O
Procura por informação atual e baseada em evidência.	O	O	O	O	O
Existência de cultura / experiência de colaboração entre associações e instituições públicas.	O	O	O	O	O
Existência de procedimentos / requisitos burocráticos complexos na inclusão de doentes em estudos	O	O	O	O	O
Convicção, por parte dos investigadores/médicos, de que os doentes não têm capacidade para participar em investigação.	O	O	O	O	O
Convicção, por parte das próprias organizações, de que os doentes não têm capacidade para participar em investigação.	O	O	O	O	O
Desconhecimento sobre como envolver os doentes em investigação por parte dos investigadores.	O	O	O	O	O
Reconhecimento, por parte dos diferentes atores da área de doença, da importância do envolvimento dos doentes em investigação.	O	O	O	O	O
Utilização de linguagem acessível e clara para os doentes no decorrer da investigação.	O	O	O	O	O
Carácter interventivo / participativo da organização.	O	O	O	O	O
Conhecimento da sociedade em geral sobre o tema da investigação clínica.	O	O	O	O	O
Conhecimento dos doentes e das organizações sobre o processo de investigação como um todo e sobre as diferentes etapas, em particular.	O	O	O	O	O

Como referido inicialmente, pretende-se com este estudo criar um **espaço de reflexão conjunta e construtiva sobre as diferentes oportunidades de envolvimento de doentes em investigação.**

Para tal, irá realizar-se um **encontro, até ao final da Presidência Portuguesa do Conselho da UE, no 2º trimestre deste ano**, onde se pretenderá identificar boas práticas, bem como

discutir pontos de melhoria para uma investigação mais eficaz e mais próxima das necessidades reais dos doentes.

Com o intuito de melhor preparar este momento, gostaríamos de lhe fazer algumas perguntas.

Como considera que a sua organização gostaria de estar mais envolvida na investigação clínica? (áreas, projetos ou fases de investigação, colaborações, etc.)

Que temas de investigação clínica gostaria de discutir com os demais intervenientes nesta área?

Em que áreas considera que é necessária maior capacitação das organizações de doentes para uma melhor participação e mais eficaz investigação clínica?

O que gostaria de referir quanto à sua experiência com más práticas no envolvimento de doentes em investigação? Que problemas de maior relevância consegue identificar?

Caracterização da Organização

Esta segunda parte do questionário destina-se à caracterização da sua organização.

Tipo de organização.

- [ ] Associação.

- ☐ Fundação.
- ☐ Movimento ou outra organização informal.
- ☐ Estatuto de Utilidade Pública.
- ☐ IPSS.
- ☐ ONGD.

A organização tem um Conselho Científico / Consultivo?

- ○ Sim.
- ○ Não.

Os órgãos sociais da organização incluem pessoas com a doença que representam?

	Sim	Não
Direção	○	○
Outros órgãos sociais	○	○

Tipo de doença(s) a que a organização se dedica.

- ☐ Doença(s) rara(s).
- ☐ Doença(s) não rara(s).
- ☐ Uma só patologia.
- ☐ Multi-patologia.

Ano de fundação da organização.

Orçamento anual *(tendo por referência o valor médio dos últimos 3 anos, podendo excluir-se o ano 2020, se considerar não representativo, devido à pandemia por Covid-19).*

- ○ Até 100 mil euros.

- ○ 100 mil a 250 mil euros.
- ○ 250 mil a 500 mil euros.
- ○ 500 mil a 1 milhão de euros.
- ○ 1 milhão de euros ou superior.

Número de associados.

- ○ Menos de 100 associados.
- ○ 100 a 499 associados.
- ○ 500 a 2999 associados.
- ○ 3000 ou mais associados.

Número de colaboradores regulares (estimativa).

	0	1-3	4-10	11-30	> 30
Número de colaboradores voluntários.	○	○	○	○	○
Número de colaboradores remunerados.	○	○	○	○	○

Atividades que melhor representam o trabalho da organização.

- ☐ Desenvolvimento de materiais educativos.
- ☐ Organização de grupos de apoio.
- ☐ Organização de eventos para educação de doentes e outros públicos.
- ☐ Financiamento de eventos de profissionais de saúde.
- ☐ Assistência financeira a doentes.
- ☐ Advocacy/ativismo a nível político.
- ☐ Participação em congressos.
- ☐ Publicações.
- ☐ Formação a entidades externas à organização.

- [ ] Colaboração em investigação.
- [ ] Outra(s). Qual(ais)?

Fontes preferenciais de consulta para construção de conhecimento / pensamento e a frequência com que as consulta.

	1. Nunca.	2. Com pouca frequência.	3. Com muita frequência.	4. Sempre.	re
Websites de instituições governamentais nacionais (Ministério da Saúde, Direção Geral de Saúde, etc.)	○	○	○	○	
Websites de organizações internacionais (Organização Mundial de Saúde, European Centre for Disease Prevention and Control, etc.)	○	○	○	○	
Publicações científicas.	○	○	○	○	
Notícias em jornais ou revistas.	○	○	○	○	
Telejornais.	○	○	○	○	
Pesquisa livre na Internet (Google, Youtube, Podcasts).	○	○	○	○	
Rádio.	○	○	○	○	
Participação em conferências/ workshops/ congressos.	○	○	○	○	
Universidades através do contacto com cientistas e investigadores.	○	○	○	○	
Empresas privadas (laboratórios farmacêuticos, alimentares, etc.)	○	○	○	○	
Amigos e / ou familiares.	○	○	○	○	
Profissionais de saúde.	○	○	○	○	

Muito obrigado pelo seu tempo e disponibilidade para responder a estas perguntas.

Gostaríamos de saber um pouco mais sobre a sua organização e, para tal, temos mais 8 perguntas que demoram cerca de 5 minutos a responder.

Tem disponibilidade para prosseguir um pouco mais?

- ○ Sim.
- ○ Não.

Âmbito geográfico.

- ☐ Nacional (Portugal).
- ☐ Regional.
- ☐ Local.
- ☐ Internacional.

Principais fontes de financiamento *(selecione as três principais fontes de financiamento da organização).*

- ☐ Quotas de Associados.
- ☐ Donativos Individuais / Peditório.
- ☐ Gestão de Património ou de Fundos Próprios da Organização.
- ☐ Prestação de Serviços.
- ☐ Financiamento Europeu / Internacional.
- ☐ Financiamento de Organizações Privadas (Indústria Farmacêutica, Empresas Privadas, etc.)
- ☐ Financiamento Público (Ministério da Saúde, Segurança Social, etc.)
- ☐ Consignação do IRS.
- ☐ Outros.

Perfil dos associados *(indique, também, por favor, a percentagem (estimada) de associados por tipo de situação que melhor os caracteriza).*

Pessoas com doença.	0	%
Familiares.	0	%
Profissionais de saúde.	0	%
Outros.	0	%
Total	0	%

Serviços disponibilizados aos associados/comunidade.

- ☐ Tratamentos.
- ☐ Internamento.
- ☐ Consultas clínicas.
- ☐ Consultas de nutrição.
- ☐ Exames / análises clínicas / rastreios.
- ☐ Informação sobre a doença.
- ☐ Apoio psicológico.
- ☐ Apoio assistencial.
- ☐ Apoio económico-financeiro.
- ☐ Informação sobre direitos.
- ☐ Apoio residencial.
- ☐ Protocolos para comparticipação e descontos (medicamentos, etc).
- ☐ Aluguer e disponibilização de espaços.
- ☐ Serviços prestados à comunidade (catering, lavandaria, ...)
- ☐ Outros? Quais?

Veículos de comunicação com associados e comunidade.

	Associados	Comunidade	Não usa
Página / blog na internet.	☐	☐	☐

	Associados	Comunidade	Não usa
Newsletter / boletim / jornal.	☐	☐	☐
Folhetos / brochuras.	☐	☐	☐
Email.	☐	☐	☐
Telefone.	☐	☐	☐
Twitter.	☐	☐	☐
Facebook.	☐	☐	☐
Instagram.	☐	☐	☐
Linkedin.	☐	☐	☐
Youtube.	☐	☐	☐
Whatsapp.	☐	☐	☐

A organização tem os seguintes departamentos?

	Sim - Departamento Interno.	Sim - Contratação Externa.	Não.
Departamento de Marketing.	☐	☐	☐
Departamento de Comunicação.	☐	☐	☐
Departamento de Relações Públicas.	☐	☐	☐

A sua organização participa em alguma rede ou organização Nacional / Europeia / Internacional?

	Sim.	Não.
Nacional	○	○
Europeia / Internacional	○	○

Que colaborações nacionais são mais relevantes para as atividades do dia-à-dia da sua organização? (selecione até 5 colaborações)

☐ Outras organizações de doentes.

☐ Ministério da Saúde.

- ☐ INFARMED, I.P..
- ☐ Direção-Geral de Saúde (DGS).
- ☐ Administração Central do Sistema de Saúde, I.P. (ACSS).
- ☐ Administrações Regionais de Saúde (ARS)
- ☐ Serviços Partilhados do Ministério da Saúde (SPMS)
- ☐ Academia / Centros de Investigação / Instituições de Educação.
- ☐ Sector Social / Fundações / Organizações sem fins lucrativos
- ☐ Sector Privado / Empresas / Laboratórios Farmacêuticos
- ☐ Cuidados de Saúde Primários.
- ☐ Cuidados de Saúde Hospitalares.
- ☐ Comissão de Ética para a Saúde (CES) externa.
- ☐ Instituto Nacional de Saúde Doutor Ricardo Jorge (INSA).
- ☐ Outra(s). Qual(ais)?

Fim

Muito obrigado pelo seu tempo e disponibilidade para responder a este questionário!

Como referido anteriormente, pretende-se com este estudo criar um **espaço de reflexão conjunta e construtiva sobre as diferentes oportunidades de envolvimento de doentes em investigação.** Os resultados deste questionário serão divulgados num **encontro, a realizar até ao final da Presidência, no 2º trimestre deste ano**, onde se pretenderá identificar boas práticas, bem como discutir pontos de melhoria para uma investigação mais eficaz e mais próxima das necessidades reais dos doentes.

Caso pretenda ser informado dos resultados e deseje estar presente neste encontro, preencha o formulário de inscrição aqui.

Partilhamos também algumas referências em que nos baseámos para construir este questionário.

[Etapas de Investigação e Oportunidades de Envolvimento de Doentes]
Geissler J et al. (2017), *Improving patient involvement in medicines research and development: a practical roadmap. Therapeutic Innovation and Regulatory Science*, 51:612–19.

[Níveis de Envolvimento]
Health Canada Policy Toolkit for Public Involvement in Decision Making (2000), Corporate Consultation Secretariat, Health Policy and Communications Branch Minister of Public Works and Government Services Canada.

[Estudo focado nas organizações de doentes, no contexto das doenças genéticas, de forma a avaliar a sua participação em investigação clínica]
Landy, D., Brinich, M., Colten, M. et al. (2012) *How disease advocacy organizations participate in clinical research: a survey of genetic organizations. Genet Med* 14, 223–228.

Powered by Qualtrics
