# Supplementary material for: Patient organisations’ views, motivations and experiences on patient involvement in cancer research: a pilot study in Portugal
Source: BMJ Open. 2024 Jan 24;14(1):e077444. doi: 10.1136/bmjopen-2023-077444 (PMC10824046; doi:10.1136/bmjopen-2023-077444)
Supplement: Author's manuscript [file bmjopen-2023-077444.draft_revisions.pdf]

BMJ Open

BMJ Open is committed to open peer review. As part of this commitment we make the peer review history of every article we publish publicly available.

When an article is published we post the peer reviewers' comments and the authors' responses online. We also post the versions of the paper that were used during peer review. These are the versions that the peer review comments apply to.

The versions of the paper that follow are the versions that were submitted during the peer review process. They are not the versions of record or the final published versions. They should not be cited or distributed as the published version of this manuscript.

BMJ Open is an open access journal and the full, final, typeset and author-corrected version of record of the manuscript is available on our site with no access controls, subscription charges or pay-per-view fees (http://bmjopen.bmj.com).

If you have any questions on BMJ Open's open peer review process please email info.bmjopen@bmj.com

BMJ Open

Patient Organizations' Views, Motivations and Experiences on Patient Involvement in Cancer Research - A Pilot Study in Portugal

Journal:	*BMJ Open*
Manuscript ID	bmjopen-2023-077444
Article Type:	Original research
Date Submitted by the Author:	05-Jul-2023
Complete List of Authors:	Roquette, Constança; Universidade Nova de Lisboa Nova School of Business and Economics; Champalimaud Foundation<br>Crisóstomo, Sofia; ISCTE-Instituto Universitario de Lisboa; GAT - Grupo de Ativistas em Tratamentos<br>Milagre, Tamara; EVITA – Cancro Hereditário<br>Ribeiro, Rute; Universidade Nova de Lisboa Escola Nacional de Saúde Pública<br>Pedro, Ana Rita; NOVA University Lisbon NOVA National School of Public Health, Public Health Research Centre, Comprehensive Health Research Center, CHRC, NOVA University Lisbon<br>Valente, André; Champalimaud Foundation
Keywords:	Patient Participation, Patient-Centered Care, ONCOLOGY

SCHOLARONE™
Manuscripts

I, the Submitting Author has the right to grant and does grant on behalf of all authors of the Work (as defined in the below author licence), an exclusive licence and/or a non-exclusive licence for contributions from authors who are: i) UK Crown employees; ii) where BMJ has agreed a CC-BY licence shall apply, and/or iii) in accordance with the terms applicable for US Federal Government officers or employees acting as part of their official duties; on a worldwide, perpetual, irrevocable, royalty-free basis to BMJ Publishing Group Ltd ("BMJ") its licensees and where the relevant Journal is co-owned by BMJ to the co-owners of the Journal, to publish the Work in this journal and any other BMJ products and to exploit all rights, as set out in our licence.

The Submitting Author accepts and understands that any supply made under these terms is made by BMJ to the Submitting Author unless you are acting as an employee on behalf of your employer or a postgraduate student of an affiliated institution which is paying any applicable article publishing charge ("APC") for Open Access articles. Where the Submitting Author wishes to make the Work available on an Open Access basis (and intends to pay the relevant APC), the terms of reuse of such Open Access shall be governed by a Creative Commons licence – details of these licences and which Creative Commons licence will apply to this Work are set out in our licence referred to above.

Other than as permitted in any relevant BMJ Author's Self Archiving Policies, I confirm this Work has not been accepted for publication elsewhere, is not being considered for publication elsewhere and does not duplicate material already published. I confirm all authors consent to publication of this Work and authorise the granting of this licence.

For peer review only

Patient Organizations' Views, Motivations and Experiences on Patient Involvement in Cancer Research - A Pilot Study in Portugal

ABSTRACT

Objectives: To characterize cancer-related patient organizations in Portugal and analyze their views, motivations, and experiences on patient involvement in cancer research.

Design: A multi-stakeholder group of patient representatives and researchers was formed and after a literature review, several online sessions and collaborative mind maps, a questionnaire was co-designed, and a survey of Portuguese cancer-related patient organizations' representatives conducted in May 2021, focusing on four dimensions: organization characterization, experience in cancer research; perception of the outcomes of that experience; motivations and expectations on patient involvement in research.

Results: 27 cancer-related patient organizations' representatives answered to the questionnaire (64% response rate, considering the universe of these organizations in Portugal), of which 20 have never participated in clinical research. The 7 organizations that have been involved in clinical research, participated in few stages of the research process and, mostly, with low levels of engagement. They showed "great interest" in participating in most research steps, although this is not reflected in a high perception of influence over these same steps. More than half claimed to have contributed to the increase patient recruitment and to a better understanding of informed consent by patients involved. Ensuring that research results are more aligned with the true needs of patients is the greatest motivation.

Conclusions: These findings provide a very complete overview of Portuguese cancer-related patient organizations, never done before. Most importantly, this study revealed that most Portuguese cancer-related patient organizations show great interest to be involved in different stages of clinical studies to ensure that research results are aligned with patients' needs. This questionnaire can be applied to other disease and umbrella patient organizations, at national or international levels, since it is not specific to any disease or the local context, contributing to a broader understanding of their views, motivations, and experiences on patient involvement in research.

For peer review only

STRENGTHS AND LIMITATIONS OF THIS STUDY

- This study provides the first in-depth characterization of Portuguese cancer-related patient organizations and their views, motivations, and experiences on patient involvement in cancer research.
- The knowledge generated by this study could be used to develop advocacy campaigns and public policies on patient involvement in cancer-research, which are more patient-centered and, thus, presenting better quality.
- The questionnaire developed can also be applied to any disease or umbrella patient organization, at either national or international levels, contributing to a better understanding of their views, motivations, and experiences on patient involvement in research.
- Although there was a 64% response rate, the universe of cancer-related patient organizations in Portugal is small, and the respondents' number < 30, which may compromise the statistical significance of the study, preventing from making broader conclusions.

For peer review only

INTRODUCTION

In the early 1980s (1) people with HIV joined voices and advocacy efforts to be involved at all levels of decision-making that affected them, from policymaking to research decisions (2,3), including identification of gaps and priorities, design, planning and conduct of research itself, as well as decisions on access to experimental medicines and subsequent regulatory authorization procedures (4). This call for meaningful involvement in decision-making was institutionalized under the motto “nothing about us, without us” (3), which was by the same time also adopted by the disability rights movement (5) and some years later appropriated by social movements in other disease areas, including cancer, Alzheimer disease, mental health, and many others. In a recent Eurobameter survey, “most respondents agreed (61%) that involving nonscientists in research and technological development ensures that science and technology respond to the needs, values and expectations of society” (6).

Patient involvement in research and care in general is key to closing the gap between beliefs and perception from researchers, care professionals and other decision-makers and the true needs and preferences of those affected (7–9). By contributing to optimize research and care, patient involvement leads to more successful responses and consequently improved outcomes that matter and make a difference to people - the so-called people-centered research and care (10), while strengthening health governance and responsiveness, and improving the quality of decision-making and facilitating its implementation. Patient organizations have been playing a key role in giving voice to patients’ needs and building collaborative networks between patient advocates, researchers, clinicians, and industry (11,12).

Even though there has been an increased interest and research on involvement frameworks and benefits, and practical recommendations have been issued by various stakeholders, patient involvement in clinical research is not yet a standard, meaningful and systematic practice (13–16). To address this, on October 13th, 2020, Germany, Portugal, and Slovenia (the trio Presidency of the Council of the European Union, at the time) signed together the *Europe: Unite against Cancer* declaration to join approaches on strengthening cancer research in Europe and to ensure that patient involvement becomes a standard practice (17). Moreover, fundamental principles for successful patient involvement in cancer research were largely discussed and determined, under a German Federal Ministry of Education and Research initiative (18).

Aligned with these initiatives and focused on the Portuguese context as a pilot project, this study aims to understand patient involvement in cancer research landscape and to identify existing good practices, as well as opportunities and challenges for improvement from a bottom-up perspective. Cancer-related patient organizations’ involvement at the different stages of medicines research and development (R&D) is characterized, based on opportunities for involvement described in the roadmap proposed by Geissler *et al.* (2017) (13). Organizations’ views, and motivations regarding patient involvement in clinical research activities are also analyzed. Lastly, descriptive analysis of the Portuguese cancer-related patient organizations is presented. Previous works that analyzed Portuguese patient organizations’ missions, activities, perspectives, and communication strategies on a large scale (19,20), did not present a particular focus on cancer-related patient organizations neither a perspective on these organization’s involvement in research and are probably outdated in face of the increasing interest and awareness on patient involvement amongst patient organizations and other stakeholders (21,22).

For peer review only - http://bmjopen.bmj.com/site/about/guidelines.xhtml

METHODS

In December 2020, encouraged by the *Europe: Unite against Cancer* declaration, a multi-stakeholder collaborative group was put in place with both patient representatives and researchers aiming to develop and conduct a survey that would provide state-of-the-art information on the opportunities and level of involvement in research of cancer-related patient organizations in Portugal, as well as, to characterize these, identify their views, motivations, and experiences. The survey development timeline is presented on **Figure 1**.

Figure 1 (supplementary file)

Questionnaire co-creation

Through the creation of an online collaborative mind map, we first identified the dimensions to be considered in the survey, as well as the variables and indicators to be included. Four main domains were consensually selected by all group members: (1) Experience in cancer research; (2) Perception of the outcomes of that experience; (3) Motivations and perspectives on patient involvement in cancer research; (4) Organization characteristics.

After several work sessions, the questionnaire structure was determined, as well as the questions and answer options that would best allow to conduct the intended analysis. The questionnaire final version has 41 questions and an approximate time of completion of 30 minutes. Most of the questions are closed-ended and multiple-choice questions. For questions about frequency, degree of influence and interest, a four-point Likert scale was used to avoid a neutral option. Open-ended questions are intended not to limit the information collected and give the respondents the opportunity to share their experience.

Categorization of opportunities for patient involvement and patient involvement level

In order to understand how Portuguese cancer-related patient organizations have been involved in research, the roadmap for patient involvement in medicines R&D proposed by Geissler *et al.* (2017) (13) was used as reference. This roadmap categorizes the R&D life cycle into four main stages and highlights 16 specific opportunities for patient involvement (**Table 1**), requiring different levels of expertise in the disease area. These opportunities were used to identify those where the surveyed organizations may have had experience with.

For peer review only

Table 1. Opportunities for patient involvement in medicines R&D adapted from Geissler et al. (2017) roadmap.

R&D stage	Opportunities for patient involvement in medicines R&D
**Research Priorities**	**Identification of patients' unmet needs**.
**Research Design and Planning**	**Study's synopsis design:** design and target population definition.
	**Protocol design:** endpoints, inclusion and exclusion criteria, quality of life measures and patient-reported outcomes, crossover, ethical and data protection issues, recruitment and dissemination plan, retention measures, risk-benefit balance, etc.
	**Practical Considerations:** contractual issues, travel expenses and support for family members.
	**Fundraising** for the research project.
	**Patient Information leaflet about the project:** content, visual design, readability, language and dissemination.
	**Informed Consent:** content, visual design, readability and language.
	**Ethical review** of the research project.
**Research Conduct and Operations**	**Investigators meetings to monitor the project:** representation of patients' perspectives on the study, recruitment, challenges, opportunities, which can trigger amendments.
	**Trial Steering Committee:** protocol follow up, improvements in patient access and adherence to the study.
	**Data and Safety Monitoring Committee:** risk / benefit assessment, drop-out issues and amendments to the project.
	**Information to participants:** communication of protocol amendments and new safety information.
	**Study reporting:** summary of interim results and dissemination in patient community.
**Dissemination, Communication, Post-approval**	**Health Technology Assessment:** assessment of value, patient-relevant outcomes and patient priorities.
	**Regulatory affairs:** preparation of lay summaries of results, information leaflets and updated safety communications.
	**Post-study communication:** contribution to publications, dissemination of research results to patient community / healthcare professionals.

Moreover, within each R&D stage and for each specific opportunity, the respondents were asked to rank their highest level of involvement. For that purpose, a scale of involvement was developed (**Table 2**) based on the adaptation of the levels of involvement proposed in the Health Canada Policy Toolkit for Public Involvement in Decision Making (2000) (23).

Table 2. Levels of involvement (adapted from the Health Canada Policy Toolkit for Public Involvement in Decision Making (2000) (23)).

Levels of Involvement	
**0**	Did not participate and/or receive any information.
**1**	Received information.
**2**	Participated in the collection of information.
**3**	Participated in the discussion of key points.
**4**	Participated in the discussion and actively decided together with other partners.
**5**	Participated as a full member of the research team, with equal decision-making power.

Target sample

The questionnaire target sample was the universe of cancer-related patient organizations' representatives in Portugal. The great majority of cancer-related patient organizations were previously identified by the co-author patient representative from *EVITA Cancro Hereditário, given that* a public comprehensive database of patient organizations does not currently exist in Portugal. This list was completed through web search to identify new organizations as well as to confirm those thought to be inactive. The final list comprised 42 cancer-related patient organizations that represent cancer patients, their caregivers and other affected and interested people, who often give wider voice to patients' needs.

Questionnaire validation

A focus group of seven patient organization's representatives was organized to test the questionnaire for content validity and comprehensiveness. Given the small size of the target sample, this focus group was constituted by non-cancer patient organization's representatives to ensure a similar context. A 3-hour session took place online, providing room for discussion and constructive feedback. The final version of the questionnaire was finalized based on the feedback collected, which proved to be valuable for the instrument improvement.

Ethics evaluation approval

The questionnaire received the approval of the Ethics Committee of the NOVA School of Business and Economics on April 7th, 2020.

Questionnaire distribution

On May 10th, 2020, the online questionnaire designed with Qualtrics software was distributed to the 42 cancer-related patient organizations' representatives via email. The objectives of the study and target audience were presented in the introduction followed by a consent request. The questionnaire was open for 2 weeks, and all the organizations were contacted by phone as a follow-up to the initial email. After the first week, all non-responders received a reminder followed by a second phone call and, finally, two days before the end date, another email was sent. Participants were not financially compensated, but the working group made a commitment to share and discuss the findings publicly and with all participants who showed interest.

For peer review only

Data handling and reporting

The respondents' data were clustered for analysis, without assessing each organization's performance being assessed separately. The data from each organization was encrypted and therefore its identity was not accessed during data analysis. Only the Principal Investigator had access to the encryption key (automatically generated by the Qualtrics software), with the sole purpose of monitoring the responses and to confirm if the organizations reached were indeed the ones on the target sample. All statistical analyses were performed with SPSS and decision-making regarding duplicates and inconsistencies in data provided by the respondents were discussed and unanimously decided in the multi-stakeholder working group.

Patient and Public Involvement

This study was ideated, designed, and conducted with and for patients since the very beginning. The multi-stakeholder research team included patient representatives from *EVITA Cancro Hereditário* (a patient organization focused on hereditary cancer) and *Mais Participação, Melhor Saúde* (a community-based action-research collaborative platform), as well as researchers from *Universidade NOVA de Lisboa* (NOVA School of Business and Economics and the Portuguese National School of Public Health) and the Champalimaud Foundation. This partnership with patient representatives and researchers contributed to a more accurate measurement instrument and more impactful study outcomes.

In June 23rd, 2020, an online forum driven by some members of the multi-stakeholder collaborative group and other patient organizations was organized - *New Partners, Better Outcomes – Excellence in Research Through Patient Engagement* - to promote a joint and constructive reflection on the different opportunities for the involvement of patients in research, as well to showcase and share experience-based insights on how partnerships between patients and researchers can help ask better questions, improve research designs, and increase the translation of research findings into the clinic. This forum brought together patient representatives in Portugal, among other healthcare stakeholders, as well as international patient advocates as Jan Geissler (Workgroup of European Cancer Patient Advocacy Networks - WECAN) and Bettina Ryll (Melanoma Patient Network Europe - MPNE), and was attended by participants and officials from Germany, Portugal and Slovenia, the countries that composed the Trio Presidency of the Council of the European Union at the time. Key highlights of the study results were briefly discussed to collect contributions for this paper.

RESULTS

Descriptive analysis of the respondent organizations

Of the 42 cancer-related patient organizations representatives that were contacted, responses were received from 27, meaning a response rate of 64%.

More than half of the respondents identified themselves as the regular representative of the organization (n=17, 63%) and characterized their organization as an "Association" (n=20, 74%) with a national scope

For peer review only

(n=20, 77%). While 10 (27%) have a Scientific Advisory Board, 21 (78%) include patients on their Direction Board. Almost half (n=12, 44%) are focused on a single cancer and 5 (19%) on a rare cancer.

There is a great heterogeneity in organizations' lifespan, the oldest having been found in 1941 and the most recent in 2020. The vast majority has an annual budget of up to 100.000€ (n=21, 78%) and 2 organizations mentioned having a budget of 1.000.000€ or more (n=2, 7%). The three most reported main sources of financing were membership fees (n=22, 85%), individual donations (n=16, 62%) and personal income tax assignment (n=12, 46%).

Regarding members, 11 (41%) have less than 100 members. Patients represent more than 60% of the organization's members for 15 (58%) of the respondents, and healthcare professionals represent 0-20% of the organization's members for 25 (96%) of the respondents. The majority (n=16, 59%) does not have any paid workers, operating exclusively based on voluntary work.

Most of the organizations use email (n=25, 96%) and telephone (n=23, 88%) to communicate with their members and 24 (92%) use their own website and Facebook to reach the general community. Twitter is not used by 21 (81%) of the organizations.

When asked about the organization's main activities, educational events for patients and other audiences (n=21, 78%), support groups (n=18, 67%) and educational materials development (n=17, 63%) were the most reported. From the 27 organizations, 13 (48%) mention having participated in scientific conferences and 7 (26%) in research publications, while 6 (22%) collaborated in research activities. Regarding services provided to members and the cancer community, information about the disease (n=23, 88%), information about patient's rights (n=23, 88%), and psychological support (n=20, 77%) were the most frequently mentioned.

Representatives were also inquired about their preferred sources for building knowledge and how often they access them. The great majority referred using "Always" or "Very frequently" the following sources: Healthcare professionals (n=26, 96%), websites of international organizations, and conferences and workshops (n=21, 78%), free Internet search (n=20, 74%), websites of national government institutions (n=19, 70%) and scientific publications (n=19, 70%). Scientists and researchers were "Rarely" or "Never" a source for building knowledge for 5 (19%) respondents.

Regarding networks and relevant collaborations, 17 (65%) of the organizations integrate a national network and 16 (62%) a European/International network. Collaboration with other patient organizations (n=18, 69%) and the private sector (companies including pharmaceutical) (n=12, 46%) are the most relevant on the day-to-day work of the organizations. Five organizations (19%) reported collaboration with academia and search centers /educational institutions as relevant for their daily work.

Experience in cancer research

Most organizations (n=20,74%) have never participated in a clinical study, the most common reason being that they were never invited to do so (n=14, 70%). Of these 20 organizations without previous experience with clinical studies, 16 (80%) also did not participate in other types of health research.

Of the 7 (26%) organizations having already participated in clinical research, 5 (71%) reported having taken the initiative to get involved and 4 (80%) of those reported having had the initiative to develop

For peer review only

the study itself. On the other side, 6 (86%) have never participated in the development of clinical recommendations/guidelines.

When enquired about the type of clinical research they were involved in, 6 (86%) mentioned having participated in observational studies, 3 (43%) in basic or pre-clinical studies and 2 (29%) in clinical trials.

Impact and outcomes of research involvement

The seven organizations with research experience were also asked about the higher level of involvement they have ever had in the different stages of the R&D life cycle as proposed by Geissler *et al*. (2017). As shown on **Figure 2,** most of the respondents reported low levels of involvement regarding the 16 different opportunities for patient involvement through the R&D life cycle. More than half of the organizations, "did not participate or did not receive information" (*Level 0*) in 10 out of the 16 opportunities for patient involvement. However, some participants reported to have been experienced at *Level 5* ("Participated as a full member of the research team, with equal decision-making power"), in activities such as: identification of the real needs of patients (n=2, 29%), study's synopsis design, fundraising for the research project and post-study communication activities (each three by n=1, 14%).

Figure 2 (supplementary file)

In regard to the agreement with different statements concerning the overall participation of the organization in the clinical studies, most respondents mention having a positive perception of that involvement**.** More than half of the organizations "Agrees" or "Strongly agrees" that: their participation has been valued by the research team during the study (n=5, 71%); their contribution has been integrated (n=5, 71%); was recognized as a partner of equal relevance in the study (n=5, 71%) and has actively participated in the research (n=4, 57%). On the other side, 3 (43%) organizations "Disagrees" or "Strongly disagrees" that, after being involved in the study, the results were shared with the organization, before being published.

Regarding the overall impact of their involvement, most respondents identified that their participation contributed to increase the patient recruitment rate (n=5, 71%), to help the patients involved with a better understanding of the study informed consent (n=4, 57%), to increase the quality of the generated knowledge and to decrease the time needed to complete the study (both n=3, 43%).

Views and motivations on patient involvement in cancer research

Regardless of whether, or not, organizations have had previous experience in participating in clinical research, all respondents were asked about their level of interest in participating in each step of the R&D life cycle and the level of influence they perceive to have in each step. As can be seen on **Figure 3**, more than half of the organizations is "Interested" or "Very interested" in being involved in all

For peer review only

different R&D life cycle steps. In particular, more than half is "Very interested" in being involved in the following phases: identification of the real needs of patients (n=20, 74%), regulatory affairs and post-study communication activities (both n=17, 63%), patient information sheet about the project (n=16, 59%), Health Technology Assessment (n=15, 56%) and informed consent (n=14, 52%). However, for the same opportunities for patient involvement in the R&D life cycle, lower levels of perceived influence were identified, as shown on **Figure 3.**

Figure 3 (supplementary file)

When respondents were asked about their motivations to be involved in clinical studies, regardless of whether they have had previous experiences, or not, 20 (74%) reported wanting to ensure that the study and its results address true patients' needs, 15 (56%) to develop health care and therapies that are more representative of patients' true needs and 13 (48%) to apply patients' experience and knowledge about their condition.

DISCUSSION

Only 26% of the respondent's Portuguese cancer-related patient organizations were ever involved in a clinical study at the time we conducted our survey, in line with previous findings from the literature (13–16), i.e., despite an increased interest and research on involvement frameworks and benefits, and the issuing of practical recommendations by various stakeholders, patient involvement in research is not yet a standard, meaningful and systematic practice.

Also, although organizations with prior research experience have a positive perception on the way they were involved and how their participation contributed to more favorable research outcomes, a low level of involvement in the various R&D steps was actually observed. This finding is corroborated by the low level of perceived influence reported by the organizations participating in the study, despite the high reported motivation to participate in the different R&D steps and the perceived benefits of the organization's involvement in research. The findings suggest that meaningful involvement in research has not yet been fully appropriated by patient organizations, neither by researchers and other stakeholders involved, and consequently not completely put into practice. Awareness raising for principles that should guide patient involvement and the benefits of patient-centered research, on top of training on the process itself, may contribute to increasing the actual level of patient involvement in the various R&D steps.

Most of the organizations, when asked about its main activities and services, identified patient's support and educational related ones as the most representative, which goes in line with the conventional understanding of a patient organization's mission and the findings of Amaro *et al,* in 2015 (19). However, it is relevant to outline from our results that five from the seven organizations involved in research reported having taken the initiative to get involved, and four of those reported having had the initiative to develop the study itself. This suggests that these organizations either are not involved in research at all or, if they do, they are the ones who promote it. Our findings do not allow us to understand *how* in fact these organizations take the initiative to develop, or to be involved, in clinical studies and

For peer review only

it would be very relevant, in future work, to understand how these partnerships between patients and researchers were initiated to provide the literature with examples of best practices and recommendations.

Moreover, when asked about *why* some organizations had never participated in a clinical study before, the most outlined reason was the fact that the organization was never invited to participate in this type of study, and this may represent a critical reason for the low involvement of these organizations in clinical studies: researchers are not reaching them. Our findings do not allow us to understand *why* and *what* is missing in the communication between patients and researchers, and therefore it would be relevant, as future work, to develop a questionnaire with mirrored questions to this one, in order to understand the researchers' side and perspective on this matter.

Regardless of having had previous experience with clinical studies, our results also reveal that most Portuguese cancer-related patient organizations have significant interest when asked about being involved in different stages of a clinical study. Ensuring that research results are more aligned with the real needs of patients are the main reported motivations. These findings are particularly relevant for researchers to be aware that these organizations are willing to collaborate in research. The motivation expressed by all organizations to participate in clinical research can and should be turned into greater and more meaningful involvement in practice, so that the cancer community can benefit from the outcomes of a truly patient-centered research.

When asked about the global impact of their participation in the studies, more than half of the seven organizations that were, or have ever been, involved in clinical studies claimed to have contributed to the increase in the participation rate of patients and a better understanding of informed consent by the patients involved. This is an important finding for the many researchers that often struggle with low retention rates in clinical studies and with the designing of an informed consent in lay language that is clear and enlightening for the patients.

Furthermore, it might be worth highlighting that the results obtained from this survey contrast significantly with the results obtained by Landy *et al.*, in 2013 (24). The latter targeted international genetic diseases advocacy organizations to understand how they participate in clinical research and found that the majority of these organizations participate directly in multiple aspects of research, ranging from study design and patient recruitment to data collection and analysis. This disparity in patient involvement experience might be due to several reasons, and, in the future, it could be relevant to analyze different factors that might have an impact on it, considering different countries and disease areas, such as the type and prevalence of a disease, participation in national and international research networks, among others.

Regarding the organizations' characterization, this study provides a very complete overview of the Portuguese cancer-related patient organizations, never conducted before, on important subjects such as: organizational structure, financial sources, members profile, activities and services, communication channels and collaborations. However, due to the small universe of Portuguese cancer-related patient organizations and the low number of organizations reporting previous involvement in clinical research, we acknowledge that the conducted descriptive analysis provides little information on the correlation of the organizations' characteristics and their involvement in clinical research. In future research it is worth further investigating the role that distancing between patient organizations and academia (universities, through contact with scientists and researchers, were "rarely" or "never" a preferred source for building knowledge by 19% of the organizations), reliance on voluntary versus paid work or

For peer review only

the role that a scientific advisory board may play, as well as been integrated in national and European/International networks.

CONCLUSIONS

This study consisted of the development and validation of a questionnaire aiming to characterize patient organizations and identify their views, motivations and experiences on patient involvement in research.

Applied to a pilot sample, consisting of Portuguese cancer-related patient organizations, our findings provide a very complete overview of these organizations, never conducted before. Most importantly, this study revealed that most Portuguese cancer-related patient organizations were never involved in research but show high interest to be involved in different stages of a clinical study and to ensure that research results are more aligned with the real needs of patients.

In the future, this questionnaire can be applied to other disease and umbrella patient organizations, at national and/or international levels, since it is not specific to any disease or the local context, contributing to a broader understanding of their and their views, motivations and experiences on patient involvement in research.

FUNDING STATEMENT

This research received no specific grant from any funding agency in the public, commercial or not-for-profit sectors.

COMPETING INTERESTS STATEMENT

The authors declare that they have no known competing financial interests or personal relationships that could have appeared to influence the work reported in this paper.

DATA AVAILABILITY STATEMENT

All data relevant to the study are included in the article or uploaded as online supplemental information.

For peer review only

REFERENCES

1. Advisory Committee of the People with AIDS. The Denver Principles [Internet]. 1983 [cited 2023 Jan 12]. Available from: https://data.unaids.org/pub/externaldocument/2007/gipa1983denverprinciples_en.pdf

2. Crimp D. Before Occupy: How AIDS Activists Seized Control of the FDA in 1988. The Atlantic [Internet]. [cited 2023 Jan 12]; Available from: https://www.theatlantic.com/health/archive/2011/12/before-occupy-how-aids-activists-seized-control-of-the-fda-in-1988/249302/

3. Lessem E. Nothing About Us Without Us: Community Engagement in Research to End the Epidemics. TAGline [Internet]. 2019 May [cited 2023 Jan 12]; Available from: https://www.treatmentactiongroup.org/wp-content/uploads/2011/08/tagline_may_2019_lessem_intro.pdf

4. Crisóstomo S, Alves da Costa F. Public Participation in Access to Medicines. In: Global Pharmaceutical Policy. Singapore: Springer Singapore; 2020. p. 261–91.

5. Charlton JI. Nothing About Us Without Us. University of California Press; 1998.

6. European Commission, Directorate-General for Research and Innovation. Special Eurobarometer 516 – April-May 2021: European citizens' knowledge and attitudes towards science and technology [Internet]. 2021. Available from: https://europa.eu/eurobarometer

7. Montgomery AA, Fahey T. How do patients' treatment preferences compare with those of clinicians? Qual Saf Health Care. 2001 Sep 1;10(Supplement 1):i39–43.

8. Sewitch MJ, Abrahamowicz M, Dobkin PL, Tamblyn R. Measuring Differences Between Patients' and Physicians' Health Perceptions: The Patient–Physician Discordance Scale. J Behav Med. 2003;26(3):245–64.

9. Harrison JD, Solomon MJ, Young JM, Meagher A, Butow P, Salkeld G, et al. Patient and Physician Preferences for Surgical and Adjuvant Treatment Options for Rectal Cancer. Archives of Surgery. 2008 Apr 1;143(4):389.

10. Report by the Secretariat of World Health Organization. Framework on integrated people-centred health services. [Internet]. 2016 [cited 2023 Jan 12]. Available from: http://apps.who.int/gb/ebwha/pdf_files/WHA69/A69_39-en.pdf?ua=1&ua=1

11. Deane K, Delbecque L, Gorbenko O, Hamoir AM, Hoos A, Nafria B, et al. Co-creation of patient engagement quality guidance for medicines development: an international multistakeholder initiative. BMJ Innov. 2019 Jan;5(1):43–55.

12. Fabbri A, Parker L, Colombo C, Mosconi P, Barbara G, Frattaruolo MP, et al. Industry funding of patient and health consumer organisations: systematic review with meta-analysis. BMJ. 2020 Jan 22;l6925.

For peer review only

13. Geissler J, Ryll B, di Priolo SL, Uhlenhopp M. Improving Patient Involvement in Medicines Research and Development: A Practical Roadmap. Ther Innov Regul Sci. 2017 Sep 30;51(5):612–9.

14. Warner K, See W, Haerry D, Klingmann I, Hunter A, May M. EUPATI Guidance for Patient Involvement in Medicines Research and Development (R&D); Guidance for Pharmaceutical Industry-Led Medicines R&D. Front Med (Lausanne). 2018 Oct 9;5.

15. Vat LE, Finlay T, Jan Schuitmaker-Warnaar T, Fahy N, Robinson P, Boudes M, et al. Evaluating the "return on patient engagement initiatives" in medicines research and development: A literature review. Health Expectations. 2020 Feb 6;23(1):5–18.

16. Cavaller-Bellaubi M, Faulkner SD, Teixeira B, Boudes M, Molero E, Brooke N, et al. Sustaining Meaningful Patient Engagement Across the Lifecycle of Medicines: A Roadmap for Action. Ther Innov Regul Sci. 2021 Sep 10;55(5):936–53.

17. Trio Presidency of the Council of the European Union, Germany, Portugal, Slovenia. Declaration on effective cancer research "Europe: Unite against Cancer" [Internet]. 2020 [cited 2023 Jan 12]. Available from: https://www.bmbf.de/SharedDocs/Downloads/en/210907-unite-against-cancer.pdf

18. German Federal Ministry of Education and Research. Principles of Successful Patient Involvement in Cancer Research [Internet]. 2021 [cited 2023 Jan 12]. Available from: https://bmbf.de/SharedDocs/Downloads/en/210907-unite-against-cancer.pdf

19. Amaro F, Pinto C, Carvalho MI. As Associações de Doentes em Portugal: Problemas, Necessidades e Aspirações. Abbvie, editor. Companhia das Ideias; 2015.

20. Bastos F. As associações de doentes em Portugal: profissionalização e estratégias de comunicação. [Internet]. ISCTE-IUL, Lisboa; 2018 [cited 2023 Jan 12]. Available from: https://repositorio.iscte-iul.pt/handle/10071/17677

21. Crisóstomo S, Santos M. Participação pública na saúde: das ideias à ação em Portugal. Rev Crit Cienc Sociais. 2018 Dec 1;(117):167–86.

22. de Freitas C. Public and patient participation in health policy, care and research. Porto Biomed J. 2017 Mar;2(2):31–2.

23. Corporate Consultation Secretariat, Health Policy and Communications Branch Minister of Public Works and Government Services Canada. Health Canada Policy Toolkit for Public Involvement in Decision Making [Internet]. 2000 [cited 2023 Jan 12]. Available from: https://www.canada.ca/content/dam/hc-sc/migration/hc-sc/ahc-asc/alt_formats/pacrb-dgapcr/pdf/public-consult/2000decision-eng.pdf

24. Landy DC, Brinich MA, Colten ME, Horn EJ, Terry SF, Sharp RR. How disease advocacy organizations participate in clinical research: a survey of genetic organizations. Genetics in Medicine. 2012 Feb;14(2):223–8.

For peer review only

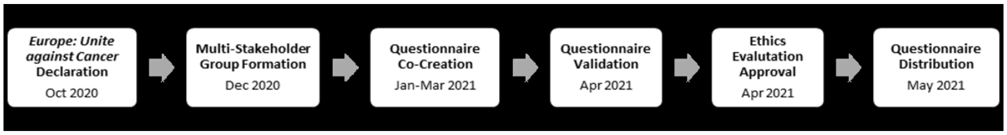

Figure 1. Survey development timeline.

790x100mm (38 x 38 DPI)

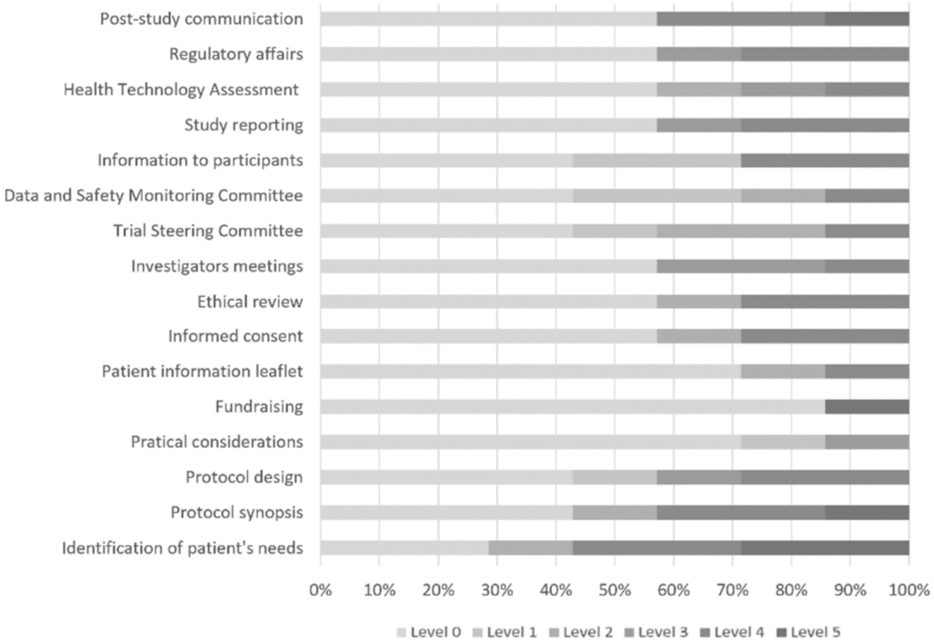

Figure 2. Respondents' higher level of involvement in the different opportunities for patient involvement during the R&D life cycle proposed by Geissler et al. (2017).

420x286mm (94 x 94 DPI)

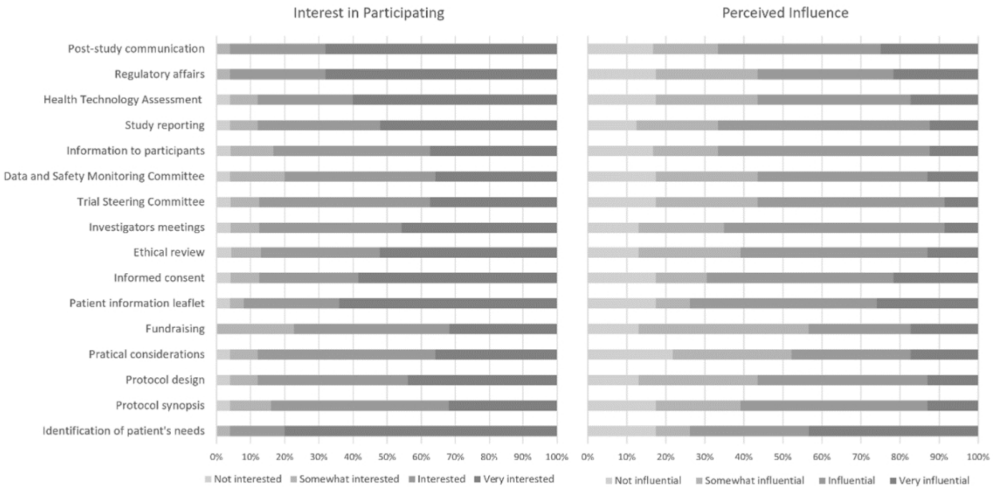

Figure 3. Respondents' interest in participating and perceived level of influence in the different opportunities for patient involvement during the R&D life cycle proposed by Geissler et al. (2017).

416x212mm (94 x 94 DPI)

Checklist for Reporting Results of Internet E-Surveys (CHERRIES)

Item Category	*Checklist Item*	*Description*
**Design**		The survey was designed with Qualtrics^{TM} software, and the target sample was the universe of cancer-related patient organizations' representatives in Portugal. The final list comprised 42 cancer-related patient organizations that represent cancer patients, their caregivers and other affected and interested people, who often give wider voice to patients' needs.
**IRB**	**IRB approval**	The questionnaire received the approval of the Ethics Committee of the NOVA School of Business and Economics on April 7th, 2020.
	**Informed consent**	On the survey welcome page, the participants were informed about the objectives of the study, the target audience, and that the questionnaire had 41 questions and an approximate time of completion of 30 minutes. A consent request was presented at the end of the introduction and participants could only access the questionnaire if they agree with it.
	**Data protection**	The respondents' data were clustered for analysis, without assessing each organization's performance being assessed separately. The data from each organization was encrypted and therefore its identity was not accessed during data analysis. Only the Principal Investigator had access to the encryption key (automatically generated by the Qualtrics software), with the sole purpose of monitoring the responses and to confirm if the organizations reached were indeed the ones on the target sample.
**Development and testing**		A focus group of seven patient organization's representatives was organized to test the survey for content validity and comprehensiveness. Given the small size of the target sample, this focus group was constituted by non-cancer patient organization's representatives to ensure a similar context. A 3-hour session took place online, providing room for discussion and constructive feedback. The final version of the survey was finalized based on the feedback collected, which proved to be valuable for the instrument improvement.
**Recruitment process**	**Open survey versus closed survey**	The survey was an open survey, distributed to the 42 cancer-related patient organizations' representatives via email.
	**Contact mode**	The survey was sent by email and stayed active for 2 weeks, and all the organizations were contacted by phone as a follow-up to the initial email. After the first week, all non-responders received a reminder followed by a second phone call and, finally, two days before the end date, another email was sent.
	**Advertising the survey**	The survey was not advertised; it was sent directly by email to the 42 cancer-related patient organizations' representatives in Portugal.
**Survey Administration**	**Web/E-mail**	This was an online survey hosted by Qualtrics^{TM}. Participants accessed the survey by clicking on the link presented on the email.

For peer review only

	**Context**	On May 10th, 2020, the online survey designed with Qualtrics™ software was distributed to the 42 cancer-related patient organizations' representatives via email.
	**Mandatory/voluntary**	Participation was voluntary, and this was outlined to participants during the survey introduction before the informed consent request.
	**Incentives**	Participants were not financially compensated, but the working group made a commitment to share and discuss the findings publicly and with all participants who showed interest.
	**Time/Date**	Responses were collected between May 10th and May 24th, 2020.
	**Randomization of items or questionnaires**	No randomization of items was used.
	**Adaptive questioning**	Adaptive questioning was used. Relevant survey items were displayed or hidden based on previous items' responses.
	**Number of Items**	The number of items per page varied between 1 – 9. The full survey comprised a total of 41 items, although because of the adaptive nature of the questionnaire, not all respondents answered all items.
	**Number of screens (pages)**	The full survey was distributed over approximately 18 pages.
	**Completeness check**	Most survey items were mandatory (outstanding items were completed by 26/27 of respondents)<br>Most items, provided a non-response option such as "not applicable" or "rather not say", and selection of one response option was enforced.
	**Review step**	A Back button was available on each page, however respondents were unable to change their responses once submitted.
**Response Rates**	**Unique site visitor**	We asked respondents to identify the organization they were representing (information only available to the Principal Investigator). Decision-making regarding duplicates and inconsistencies in data provided by the respondents were discussed and unanimously decided in the multi-stakeholder working group.
	**View rate (Ratio of unique survey visitors/unique site visitors)**	Necessary detail for calculation was not recorded.
	**Participation rate (Ratio of unique visitors who agreed to participate/unique first survey page visitors)**	Necessary detail for calculation according to the proposed formula was not recorded. However, the survey had a participation rate of 64%, since 27/42 invited patient representatives submitted a response.

For peer review only

	**Completion rate (Ratio of users who finished the survey/users who agreed to participate)**	Necessary detail for calculation was not recorded.
**Preventing multiple entries**	**Cookies used**	Cookies were not used.
	**IP check**	IP addresses were not collected.
	**Log file analysis**	The study did not include a log file analysis.
	**Registration**	Not applicable.
**Analysis**	**Handling of incomplete questionnaires**	Only completed questionnaires were included in the final dataset.
	**Questionnaires submitted with an atypical timestamp**	No atypical timestamps were detected.
	**Statistical correction**	None required.

This checklist has been modified from Eysenbach G. Improving the quality of Web surveys: the Checklist for Reporting Results of Internet E-Surveys (CHERRIES). J Med Internet Res. 2004 Sep 29;6(3):e34 [erratum in J Med Internet Res. 2012; 14(1): e8.]. Article available at https://www.jmir.org/2004/3/e34/; erratum available https://www.jmir.org/2012/1/e8/. Copyright ©Gunther Eysenbach. Originally published in the Journal of Medical Internet Research, 29.9.2004 and 04.01.2012.

This is an open-access article distributed under the terms of the Creative Commons Attribution License (https://creativecommons.org/licenses/by/2.0/), which permits unrestricted use, distribution, and reproduction in any medium, provided the original work, first published in the Journal of Medical Internet Research, is properly cited.

BMJ Open

Patient Organizations' Views, Motivations and Experiences on Patient Involvement in Cancer Research - A Pilot Study in Portugal

Journal:	*BMJ Open*
Manuscript ID	bmjopen-2023-077444.R1
Article Type:	Original research
Date Submitted by the Author:	22-Nov-2023
Complete List of Authors:	Roquette, Constança; Universidade Nova de Lisboa Nova School of Business and Economics<br>Crisóstomo, Sofia; ISCTE-Instituto Universitario de Lisboa; GAT - Grupo de Ativistas em Tratamentos<br>Milagre, Tamara; EVITA – Cancro Hereditário<br>Ribeiro, Rute; Universidade Nova de Lisboa Escola Nacional de Saúde Pública<br>Pedro, Ana Rita; NOVA University Lisbon NOVA National School of Public Health, Public Health Research Centre, Comprehensive Health Research Center, CHRC, NOVA University Lisbon<br>Valente, André; Champalimaud Foundation
**Primary Subject Heading**:	Patient-centred medicine
Secondary Subject Heading:	Oncology, Patient-centred medicine
Keywords:	Patient Participation, Patient-Centered Care, ONCOLOGY

SCHOLARONE™
Manuscripts

I, the Submitting Author has the right to grant and does grant on behalf of all authors of the Work (as defined in the below author licence), an exclusive licence and/or a non-exclusive licence for contributions from authors who are: i) UK Crown employees; ii) where BMJ has agreed a CC-BY licence shall apply, and/or iii) in accordance with the terms applicable for US Federal Government officers or employees acting as part of their official duties; on a worldwide, perpetual, irrevocable, royalty-free basis to BMJ Publishing Group Ltd ("BMJ") its licensees and where the relevant Journal is co-owned by BMJ to the co-owners of the Journal, to publish the Work in this journal and any other BMJ products and to exploit all rights, as set out in our licence.

The Submitting Author accepts and understands that any supply made under these terms is made by BMJ to the Submitting Author unless you are acting as an employee on behalf of your employer or a postgraduate student of an affiliated institution which is paying any applicable article publishing charge ("APC") for Open Access articles. Where the Submitting Author wishes to make the Work available on an Open Access basis (and intends to pay the relevant APC), the terms of reuse of such Open Access shall be governed by a Creative Commons licence – details of these licences and which Creative Commons licence will apply to this Work are set out in our licence referred to above.

Other than as permitted in any relevant BMJ Author's Self Archiving Policies, I confirm this Work has not been accepted for publication elsewhere, is not being considered for publication elsewhere and does not duplicate material already published. I confirm all authors consent to publication of this Work and authorise the granting of this licence.

For peer review only

Patient Organizations' Views, Motivations and Experiences on Patient Involvement in Cancer Research - A Pilot Study in Portugal

Constança Roquette[1], Sofia Crisóstomo[2,3], Tamara Milagre[4], Rute Simões Ribeiro[5], Ana Rita Pedro[6], André Valente[7]

1. Nova School of Business and Economics, Nova University Lisbon, Lisbon, Portugal
2. ISCTE - University Institute of Lisbon, Lisbon, Portugal
3. GAT - *Grupo de Ativistas em Tratamentos*, Lisbon, Portugal
4. EVITA – *Cancro Hereditário*, Lisbon, Portugal
5. Nova National School of Public Health, Nova University Lisbon, Lisbon, Portugal
6. Public Health Research Centre, Comprehensive Health Research Center, CHRC, Nova National School of Public Health, Nova University Lisbon, Lisbon, Portugal
7. Champalimaud Foundation, Lisbon, Portugal

Corresponding Author:
Name: Constança Roquette
Address: Nova School of Business and Economics, R. da Holanda 1, 2775-405 Carcavelos, Portugal
Email: constanca.roquette@novasbe.pt
Telephone: +351 96 306 84 10

Keywords: Patient Participation, Community-Based Participatory Research, Patient-Centred Medicine, Oncology

Word count: 5793 (excluding title page, references, figures); 5223 (excluding abstract, strengths, and limitations, statements, and references)

For peer review only

ABSTRACT

Objectives: To characterize Portuguese cancer-related patient organizations and analyze their views, motivations, and experiences on patient involvement in cancer research.

Design: A multi-stakeholder group, comprising patient representatives and researchers, co-designed a questionnaire after a literature review, online sessions, and collaborative mind maps. In May 2021, a survey was conducted among representatives of Portuguese cancer-related patient organizations, focusing on four dimensions: experience in cancer research; perception of its outcomes; motivations and expectations on patient involvement in research; and organization characteristics.

Participants: Twenty-seven representatives from Portuguese cancer-related patient organizations responded to the questionnaire, corresponding to a 64% response rate.

Results: Among the surveyed organizations, 26% have participated in clinical studies. Their involvement occurred in few stages of the research process and, mostly, with low levels of engagement. They showed "great interest" in participating in most research steps, although this is not reflected in a high perception of influence over these same steps. More than half claimed to have contributed to the increase in patient recruitment and to a better understanding of informed consent by patients involved. Ensuring that research results are more aligned with the true needs of patients is the greatest motivation. Also, our results suggest that the organization's number of employees and its integration into a European/International network play a relevant role in patient involvement in research.

Conclusions: This study provides the first in-depth characterization of Portuguese cancer-related patient organizations and their views, motivations, and experiences on patient involvement in cancer research. Most importantly, this study revealed that most of these organizations show great interest in being involved in different R&D stages to ensure that research results are aligned with patients' needs. Their motivation should be turned into greater and more meaningful involvement in practice, so that the cancer community can benefit from the outcomes of truly patient-centered research.

For peer review only

STRENGTHS AND LIMITATIONS OF THIS STUDY

- A four-domain questionnaire was developed to characterize Portuguese cancer-related patient organizations and their views, motivations, and experiences regarding patient involvement in cancer research.
- This research methodically investigates the level of patient involvement in 16 R&D opportunities, going beyond previous studies that mainly focus on identifying the presence or absence of patient involvement through the R&D process.
- This study was a multistakeholder co-creation, having been ideated, designed, and conducted with and for patients since the very beginning.
- Although there was a 64% response rate, the universe of cancer-related patient organizations in Portugal is small, and the respondents' number < 30, which may compromise the statistical significance of the study, preventing from making broader conclusions.

For peer review only

INTRODUCTION

In the early 1980s (1) people with HIV joined voices and advocacy efforts to be involved at all levels of decision-making that affected them, from policymaking to research decisions (2,3), including identification of gaps and priorities, design, planning and conduct of research itself, as well as decisions on access to experimental medicines and subsequent regulatory authorization procedures (4). This call for meaningful involvement in decision-making was institutionalized under the motto "nothing about us, without us" (3), which was by the same time also adopted by the disability rights movement (5) and some years later appropriated by social movements in other disease areas, including cancer, Alzheimer disease, mental health, and many others. In a recent Eurobarometer survey, "most respondents agreed (61%) that involving nonscientists in research and technological development ensures that science and technology respond to the needs, values and expectations of society" (6).

Patient involvement in research and care in general is key to closing the gap between beliefs and perception from researchers, care professionals and other decision-makers and the true needs and preferences of those affected (7–9). By contributing to optimize research and care, patient involvement leads to more successful responses and consequently improved outcomes that matter and make a difference to people, the so-called people-centered research and care (10), while strengthening health governance and responsiveness, and improving the quality of decision-making and facilitating its implementation. Patient organizations have been playing a key role in giving voice to patients' needs while building collaborative networks between patient advocates, researchers, clinicians, and industry, and steering research and development (R&D) activity, particularly in the context of rare diseases (11–13).

Even though there has been an increased interest and research on involvement frameworks and benefits, and practical recommendations have been issued by various stakeholders, patient involvement in clinical research is not yet a standard, meaningful and systematic practice (14–23) and several challenges are identified. There is a lack of clarity of the concept of "patient involvement", which leads to challenges for operationalizing and measuring its impacts (24), and very often involvement is mostly done through consultation, instead of direct engagement, being rarely extended to later stages of the R&D process (25–27). Matching patient advocates with research opportunities for involvement is also challenging, particularly when the dissemination of these opportunities is limited (28,29). Finally, there is a lack of or fragmented reporting and critical reflection on patient involvement activities (27,29), which calls for more research that compares, contrasts and evaluates different patient involvement initiatives (30).

To address this, on October 13th, 2020, Germany, Portugal, and Slovenia (the trio Presidency of the Council of the European Union, at the time) signed together the *Europe: Unite against Cancer* declaration to join approaches on strengthening cancer research in Europe and to ensure that patient involvement becomes a standard practice (31). Moreover, fundamental principles for successful patient involvement in cancer research were largely discussed and determined, under a German Federal Ministry of Education and Research initiative (32).

Aligned with these initiatives and focused on the Portuguese context as a pilot project, this study aims to understand patient involvement in cancer research landscape and to identify existing good practices, as well as opportunities and challenges for improvement from a bottom-up perspective. Cancer-related patient organizations' involvement at the different stages of medicines R&D is characterized, based on opportunities for involvement described in the roadmap proposed by Geissler *et al.* (2017) (14).

For peer review only

Organizations' views, and motivations regarding patient involvement in clinical research activities are also analyzed. Lastly, descriptive analysis of the Portuguese cancer-related patient organizations is presented. Previous works that analyzed Portuguese patient organizations' missions, activities, perspectives, and communication strategies on a large scale (33,34), did not present a particular focus on cancer-related patient organizations neither a perspective on these organization's involvement in research and are probably outdated in face of the increasing interest and awareness on patient involvement amongst patient organizations and other stakeholders (35,36).

METHODS

In December 2020, encouraged by the *Europe: Unite against Cancer* declaration, a multi-stakeholder collaborative group with 6 people was put in place with both patient representatives and researchers, aiming to develop and conduct a survey that would provide state-of-the-art information on the opportunities and level of involvement in research of cancer-related patient organizations in Portugal, as well as, to characterize these, identify their views, motivations, and experiences. The multi-stakeholder research team included two patient advocates, one from *EVITA Cancro Hereditário* (a patient organization focused on hereditary cancer) and another from *Mais Participação, Melhor Saúde* (a community-based action-research collaborative platform), as well as three researchers from *Universidade NOVA de Lisboa* (NOVA School of Business and Economics and the Portuguese National School of Public Health) and one researcher from the Champalimaud Foundation. The survey development timeline is presented in **Figure 1**.

Figure 1 (supplementary file)

Questionnaire co-creation

Through the creation of an online collaborative mind map, we first identified the dimensions to be considered in the survey, as well as the variables and indicators to be included. Four main domains were consensually selected by all group members: (1) Experience in cancer research; (2) Perception of the outcomes of that experience; (3) Motivations and perspectives on patient involvement in cancer research; (4) Organization characteristics.

After several work sessions, the questionnaire structure was agreed, as well as the questions and answer options that would best allow to conduct the intended analysis. The questionnaire final version has 41 questions, an approximate time of completion of 30 minutes, and is attached as a supplementary file (questionnaire Portuguese version and English translation files). Most of the questions are closed-ended and multiple-choice questions. For questions about frequency, degree of influence and interest, a four-point Likert scale was used to avoid a neutral option. Open-ended questions are intended not to limit the information collected and give the respondents the opportunity to share their experience.

Categorization of opportunities for patient involvement and patient involvement level

In order to understand how Portuguese cancer-related patient organizations have been involved in research, the roadmap for patient involvement in medicines R&D proposed by Geissler *et al.* (2017) (14) was used as reference. This roadmap categorizes the R&D life cycle into four main stages and

For peer review only

highlights 16 specific opportunities for patient involvement (**Table 1**), requiring different levels of expertise in the disease area. These opportunities were used to identify those where the surveyed organizations may have had experience with.

Table 1. Opportunities for patient involvement in medicines R&D adapted from Geissler et al. (2017) roadmap.

R&D stage	Opportunities for patient involvement in medicines R&D
**Research Priorities**	**Identification of patients' unmet needs**.
**Research Design and Planning**	**Study's synopsis design:** design and target population definition.
	**Protocol design:** endpoints, inclusion and exclusion criteria, quality of life measures and patient-reported outcomes, crossover, ethical and data protection issues, recruitment and dissemination plan, retention measures, risk-benefit balance, etc.
	**Practical Considerations:** contractual issues, travel expenses and support for family members.
	**Fundraising** for the research project.
	**Patient Information leaflet about the project:** content, visual design, readability, language and dissemination.
	**Informed Consent:** content, visual design, readability and language.
	**Ethical review** of the research project.
**Research Conduct and Operatio ns**	**Investigators meetings to monitor the project:** representation of patients' perspectives on the study, recruitment, challenges, opportunities, which can trigger amendments.
	**Trial Steering Committee:** protocol follow up, improvements in patient access and adherence to the study.
	**Data and Safety Monitoring Committee:** risk / benefit assessment, drop-out issues and amendments to the project.
	**Information to participants:** communication of protocol amendments and new safety information.
	**Study reporting:** summary of interim results and dissemination in patient community.
**Dissemin ation, Commun ication, Post-approval**	**Health Technology Assessment:** assessment of value, patient-relevant outcomes and patient priorities.
	**Regulatory affairs:** preparation of lay summaries of results, information leaflets and updated safety communications.
	**Post-study communication:** contribution to publications, dissemination of research results to patient community / healthcare professionals.

Moreover, within each R&D stage and for each specific opportunity, the respondents were asked to rank their highest level of involvement. For that purpose, a scale of involvement was developed (**Table 2**) based on the adaptation of the levels of involvement proposed in the Health Canada Policy Toolkit for Public Involvement in Decision Making (2000) (37).

Table 2. Levels of involvement (adapted from the Health Canada Policy Toolkit for Public Involvement in Decision Making (2000) (37)).

Levels of Involvement	
**0**	Did not participate and/or receive any information.
**1**	Received information.
**2**	Participated in the collection of information.
**3**	Participated in the discussion of key points.
**4**	Participated in the discussion and actively decided together with other partners.
**5**	Participated as a full member of the research team, with equal decision-making power.

Target sample and participants' eligibility criteria

The study focused on representatives from cancer-related patient organizations in Portugal as the target sample. Identification of these organizations was primarily conducted by the co-author patient representative from *EVITA Cancro Hereditário*, as there is currently no comprehensive public database of patient organizations in Portugal. The initial list was supplemented through a web search to identify new organizations and verify the status of those believed to be inactive. The final sample included 42 cancer-related patient organizations, representing individuals such as cancer patients, caregivers, and other stakeholders who actively advocate for patients' needs.

Questionnaire validation

Extensive literature review and consideration of theoretical frameworks were undertaken to identify key dimensions associated with patient's involvement in research. The questionnaire items were thoroughly selected to align with these dimensions, ensuring a comprehensive coverage of the intended construct. Emphasis was placed on clarity, relevance, and representativeness of the items to guarantee the questionnaire's effectiveness.

To test the questionnaire's face validity and comprehensiveness, a focus group of seven patient organization's representatives was organized. Given the small size of the target sample, this focus group was constituted by non-cancer patient organization's representatives to ensure a similar context. A 3-hour session took place online, providing room for discussion and constructive feedback. The final version of the questionnaire was finalized based on the feedback collected, which proved to be valuable for the instrument improvement.

Ethics evaluation approval

This study, including the questionnaire, received the approval of the Ethics Committee of the NOVA School of Business and Economics on April 7th, 2020.

Questionnaire distribution

On May 10th, 2020, the online questionnaire designed with Qualtrics software was distributed to the 42 cancer-related patient organizations' representatives via email. The objectives of the study and target audience were presented in the introduction followed by a consent request. The questionnaire was open for 2 weeks, and all the organizations were contacted by phone as a follow-up to the initial email. After the first week, all non-responders received a reminder followed by a second phone call and, finally, two

For peer review only

days before the end date, another email was sent. Participants were not financially compensated, but the working group made a commitment to share and discuss the findings publicly with all participants who showed interest.

Data handling and reporting

The respondents' data were clustered for analysis, and the identity of each organization was encrypted and not accessed during data analysis. Only the Principal Investigator had access to the encryption key (automatically generated by the Qualtrics software), with the sole purpose of monitoring the responses and to confirm if the organizations reached were indeed the ones on the target sample. All statistical analyses were performed with IBM SPSS Statistics (version 29.0) and decision-making regarding duplicates and inconsistencies in data provided by the respondents were discussed and unanimously decided in the multi-stakeholder working group.

Patient and Public Involvement

As previously mentioned, this study was ideated, designed, and conducted with and for patients since the very beginning. The multi-stakeholder research team included two patient advocates, one from *EVITA Cancro Hereditário*, a patient organization focused on hereditary cancer, and another from *Mais Participação, Melhor Saúde*, a community-based action-research collaborative platform.

In June 23rd, 2020, an online forum driven by some members of the multi-stakeholder collaborative group and other patient organizations was organized – *New Partners, Better Outcomes – Excellence in Research Through Patient Engagement* – to promote a joint and constructive reflection on the different opportunities for the involvement of patients in research, as well to showcase and share experience-based insights on how partnerships between patients and researchers can help ask better questions, improve research designs, and increase the translation of research findings into the clinic. This forum brought together patient representatives in Portugal, among other healthcare stakeholders, as well as international patient advocates as Jan Geissler (Workgroup of European Cancer Patient Advocacy Networks – WECAN) and Bettina Ryll (Melanoma Patient Network Europe – MPNE), and was attended by participants and officials from Germany, Portugal and Slovenia, the countries that composed the Trio Presidency of the Council of the European Union at the time. Key highlights of the study results were briefly discussed to collect contributions for this paper.

Data analysis

Data were analyzed using IBM SPSS Statistics (version 29.0). Statistical analysis included: i) descriptive statistics (absolute and relative frequencies); ii) Chi-square test of independence for discrete variables or Fisher's exact test of independence, in case of expected frequencies less than 5 in 2x2 contingency tables, to investigate the potential correlation between patient organisation characteristics and their involvement in clinical research; and iii). Cronbach's alpha coefficient and average inter-item correlation, to measure the internal consistency (reliability) of the set of survey items related with the organizations' interest to participate and their perceived influence in relation to the 16 R&D opportunities considered. A significance level of 0.05 and 0.001 was used for all statistical tests.

For peer review only

RESULTS

Descriptive analysis of the respondent organizations

Of the 42 cancer-related patient organizations representatives that were contacted, responses were received from 27, meaning a response rate of 64%. A brief summary of the characteristics of these organizations is presented in **Table 3**.

Table 3. Summary of the characteristics of the 27 Portuguese cancer-related patient organizations that responded to the questionnaire.

**Characteristic**		**N (%)**
Scientific Advisory Board		
Yes		10 (37.0%)
Direction Board		
With patient members		21 (77.8%)
Associate Members		
< 100 members		11 (40.7%)
100 - 499 members		5 (18.5%)
500 - 2999 members		8 (29.6%)
>= 3000 members		3 (11.1%)
Annual budget		
< 100 thousand euros		21 (77.8%)
100 - 250 thousand euros		3 (11.1%)
250 - 500 thousand euros		1 (3.7%)
500 - 1 million euros		0 (0.0%)
> 1 million euros		2 (7.4%)
Regular collaborators	*Volunteers*	*Employees*
0	1 (3.7%)	16 (59.2%)
1-3	7 (25.9%)	6 (22.2%)
4-10	11 (40.7%)	2 (7.4%)
11-30	3 (11.1%)	2 (7.4%)
> 30	5 (18.5%)	1 (3.7%)
Collaborative Networks		
National		17 (70%)
European / International		16 (64%)

More than half of the respondents identified themselves as the regular representative of the organization (n=17, 63%). While 10 (37%) have a Scientific Advisory Board, 21 (78%) include patients on their Direction Board and almost half (n=12, 44%) are focused on a single cancer and 5 (19%) on a rare cancer.

There is a great heterogeneity in organizations' lifespan, the oldest having been found in 1941 and the most recent in 2020. The vast majority have an annual budget of up to 100.000€ (n=21, 78%) and 2 organizations mentioned having a budget of 1.000.000€ or more (n=2, 7%). The three most reported main sources of financing were membership fees (n=22, 85%), individual donations (n=16, 62%) and personal income tax assignment (n=12, 46%).

Regarding members, 11 (41%) have less than 100 members. Patients represent more than 60% of the organization's members for 15 (58%) of the respondents, and healthcare professionals represent 0-20%

of the organization's members for 25 (96%) of the respondents. The majority (n=16, 59%) does not have any paid workers, operating exclusively based on voluntary work.

Most of the organizations use email (n=25, 96%) and telephone (n=23, 88%) to communicate with their members and 24 (92%) use their own website and Facebook to reach the general community. Twitter is not used by 21 (81%) of the organizations.

When asked about the organization's main activities, educational events for patients and other audiences (n=21, 78%), support groups (n=18, 67%) and educational materials development (n=17, 63%) were the most reported. Of the 27 organizations, 13 (48%) mention having participated in scientific conferences and 7 (26%) in research publications, while 6 (22%) collaborated in research activities. Regarding services provided to members and the cancer community, information about the disease (n=23, 88%), information about patient's rights (n=23, 88%), and psychological support (n=20, 77%) were the most frequently mentioned.

Representatives were also inquired about their preferred sources for building knowledge and how often they access them. The great majority referred using "Always" or "Very frequently" the following sources: Healthcare professionals (n=26, 96%), websites of international organizations, and conferences and workshops (n=21, 78%), free Internet search (n=20, 74%), websites of national government institutions (n=19, 70%) and scientific publications (n=19, 70%). Scientists and researchers were "Rarely" or "Never" a source for building knowledge for 5 (19%) respondents.

Regarding networks and relevant collaborations, 17 (70%) of the organizations integrate a national network and 16 (64%) a European/International network. Collaboration with other patient organizations (n=18, 69%) and the private sector (companies including pharmaceutical) (n=12, 46%) are the most relevant on the day-to-day work of the organizations. Five organizations (19%) reported collaboration with academia and search centers /educational institutions as relevant for their daily work.

Experience in cancer research

Most organizations (n=20,74%) have never participated in a clinical study, the most common reason being that they were never invited to do so (n=14, 70%). Of these 20 organizations without previous experience with clinical studies, 16 (80%) also did not participate in other types of health research, such as epidemiological and public health studies, public health.

Of the 7 (26%) organizations having already participated in clinical research, 5 (71%) reported having taken the initiative to get involved and 4 (80%) of those reported having had the initiative to develop the study itself. On the other side, 6 (86%) have never participated in the development of clinical recommendations/guidelines.

When enquired about the type of clinical research they were involved in, 6 (86%) mentioned having participated in observational studies, 3 (43%) in basic or pre-clinical studies and 2 (29%) in clinical trials.

Two organizational characteristics were significantly correlated with patient organization involvement in clinical research: number of employees (p=0.005) and membership of a European/International network (p=0.035). No correlation (p>0.05) was found for other organizational characteristics (having

For peer review only

a scientific advisory board, presence of patients in the direction board, number of associate members, annual budget and membership of a national network).

Impact and outcomes of research involvement

The seven organizations with research experience were also asked about the higher level of involvement they have ever had in the different stages of the R&D life cycle as proposed by Geissler *et al*. (2017). As shown on **Figure 2,** most of the respondents reported low levels of involvement regarding the 16 different opportunities for patient involvement through the R&D life cycle. More than half of the organizations, "did not participate or did not receive information" (*Level 0*) in 10 out of the 16 opportunities for patient involvement. However, some participants reported to have been experienced at *Level 5* ("Participated as a full member of the research team, with equal decision-making power"), in activities such as: identification of the real needs of patients (n=2, 29%), study's synopsis design, fundraising for the research project and post-study communication activities (each three by n=1, 14%).

Figure 2 (supplementary file)

In regard to the agreement with different statements concerning the overall participation of the organization in the clinical studies, most respondents mention having a positive perception of that involvement**.** More than half of the organizations "Agrees" or "Strongly agrees" that: their participation has been valued by the research team during the study (n=5, 71%); their contribution has been integrated (n=5, 71%); was recognized as a partner of equal relevance in the study (n=5, 71%) and has actively participated in the research (n=4, 57%). On the other side, 3 (43%) organizations "Disagrees" or "Strongly disagrees" that, after being involved in the study, the results were shared with the organization, before being published.

Regarding the overall impact of their involvement, most respondents identified that their participation contributed to increase the patient recruitment rate (n=5, 71%), to help the patients involved with a better understanding of the study informed consent (n=4, 57%), to increase the quality of the generated knowledge and to decrease the time needed to complete the study (both n=3, 43%).

Views and motivations on patient involvement in cancer research

Regardless of whether, or not, organizations have had previous experience in participating in clinical research, all respondents were asked about the level of interest of their organizations in participating in each step of the R&D life cycle and the level of influence they perceived to have in each step. Results are in **Figure 3**. More than half of the organizations is "Interested" or "Very interested" in being involved in any of the different R&D life cycle steps. For the set of 16 steps, Cronbach's alpha was 0.929 and average inter-item correlation was 0.461, indicating an acceptable internal consistency. In particular, more than half is "Very interested" in being involved in the following phases: identification of the real needs of patients (n=20, 74%), regulatory affairs and post-study communication activities (both n=17, 63%), patient information sheet about the project (n=16, 59%), Health Technology Assessment (n=15, 56%) and informed consent (n=14, 52%). However, for the same opportunities for

patient involvement in the R&D life cycle, lower levels of perceived influence were identified and Cronbach's alpha was 0.987 and average inter-item correlation was 0.830, suggesting that the items are highly inter correlated and hence redundant.

Figure 3 (supplementary file)

When respondents were asked about their motivations to be involved in clinical studies, regardless of whether they have had previous experiences, or not, 20 (74%) reported wanting to ensure that the study and its results address true patients' needs, 15 (56%) to develop health care and therapies that are more representative of patients' true needs and 13 (48%) to apply patients' experience and knowledge about their condition.

DISCUSSION

Only 26% of the respondent's Portuguese cancer-related patient organizations were ever involved in a clinical study at the time we conducted our survey, in line with previous findings from the literature (14–17), i.e., despite an increased interest and research on involvement frameworks and benefits, and the issuing of practical recommendations by various stakeholders, patient involvement in research is not yet a standard, meaningful and systematic practice.

Also, although organizations with prior research experience have a positive perception on the way they were involved and how their participation contributed to more favorable research outcomes, a low level of involvement in the various R&D steps was actually observed. This finding is corroborated by the low level of perceived influence reported by the organizations participating in the study, despite the high reported motivation to participate in the different R&D steps and the perceived benefits of the organization's involvement in research. The findings suggest that meaningful involvement in research has not yet been fully appropriated by patient organizations, neither by researchers and other stakeholders involved, and consequently not completely put into practice. The gap analysis in patient involvement practices conducted by Faulkner *et al.* 2021 (29) lead to clear directional insights to enhance collaborative practices that might mitigate this, such as empowerment of stakeholders (both patients and researchers) focusing on training on their roles and responsibilities. Furthermore, awareness raising for principles that should guide patient involvement and the benefits of patient-centered research, may contribute to increasing the actual level of patient involvement in the various R&D steps.

Most of the organizations, when asked about its main activities and services, identified patient's support and educational related ones as the most representative, which goes in line with the conventional understanding of a patient organization's mission and the findings of Amaro *et al,* in 2015 (33). However, it is relevant to outline from our results that five of the seven organizations involved in research reported having taken the initiative to get involved, and four of those reported having had the initiative to develop the study itself. This suggests that these organizations either are not involved in research at all or, if they do, they are the ones who promote it. Our findings do not allow us to understand *how* in fact these organizations take the initiative to develop, or to be involved, in clinical studies and it would be very relevant, in future work, to understand how these partnerships between patients and

For peer review only

researchers were initiated to provide the literature with examples of best practices and recommendations.

Moreover, when asked about *why* some organizations had never participated in a clinical study before, the most outlined reason was the fact that the organization was never invited to participate in this type of study, and this may represent a critical reason for the low involvement of these organizations in clinical studies: researchers are not reaching them. Our findings do not allow us to understand *why* and *what* is missing in the communication between patients and researchers, and therefore it would be relevant, as future work, to understand how these organizations could make their interest in being partners in research projects more visible and clearer, and what is preventing them from having a more active role in taking the initiative to be involved in research. The lack of fully dedicated and professionalized teams, as our results suggest, may play a relevant role in this. Also, developing a questionnaire with mirrored questions to this one, addressed to researchers would be essential to understand the researchers' side and perspective on this matter.

Regardless of having had previous experience with clinical studies, our results also reveal that most Portuguese cancer-related patient organizations have significant interest when asked about being involved in different stages of a clinical study. Ensuring that research results are more aligned with the real needs of patients are the main reported motivations. These findings are particularly relevant for researchers to be aware that these organizations are willing to collaborate in research. The motivation expressed by all organizations to participate in clinical research can and should be turned into greater and more meaningful involvement in practice, so that the cancer community can benefit from the outcomes of a truly patient-centered research.

When asked about the global impact of their participation in the studies, more than half of the seven organizations that were, or have ever been, involved in clinical studies claimed to have contributed to the increase in the participation rate of patients and a better understanding of informed consent by the patients involved. This is an important finding for the many researchers that often struggle with low retention rates in clinical studies and with the designing of an informed consent in lay language that is clear and enlightening for the patients.

Furthermore, it might be worth highlighting that the results obtained from this survey contrast significantly with the results obtained by Landy *et al.*, in 2013 (38). The latter targeted international genetic diseases advocacy organizations to understand how they participate in clinical research and found that the majority of these organizations participate directly in multiple aspects of research, ranging from study design and patient recruitment to data collection and analysis. However, our results are more aligned with the findings of Halvorsrud *et al.* 2021 (26) and Pii *et al.* 2019 (27), where evidence showed that co-creation was rarely extended to later stages of research and public and patient involvement in cancer research has especially integrated in the early stages of the research process, in defining and prioritizing research. This disparity in patient involvement through the R&D life cycle might be due to several reasons, and, in the future, it could be relevant to analyze different factors that might have an impact on it, considering different countries and disease areas, such as the type and prevalence of a disease. Our results suggest that participation in European/international research networks is a factor that may play a role in boosting patient involvement in cancer research.

Unlike earlier studies, a key aspect of our research is the in-depth examination of patient involvement across different stages of the R&D process. While prior studies focused on identifying whether patients are part of different R&D steps, our study also analyzed the extent of their participation. This

For peer review only

methodological refinement contributes to a more profound comprehension of how patients participated throughout the R&D continuum.

Regarding the organizations' characterization, this study provides a very complete overview of the Portuguese cancer-related patient organizations, never conducted before, on important subjects such as: organizational structure, financial sources, members profile, activities and services, communication channels and collaborations. However, due to the small universe of Portuguese cancer-related patient organizations and the low number of organizations reporting previous involvement in clinical research, we acknowledge that the conducted descriptive analysis provides little information on the correlation of the organizations' characteristics and their involvement in clinical research. Our analysis highlights that the number of employees (paid collaborators) and integration in a European/International network are factors that may play a role on the higher involvement of patients in research. The first factor may contribute to the necessary infrastructure in support of involvement in research activities, and the latter may enable the organization to become more visible to potential partners and more aware of opportunities for involvement. In future research, it is worth further investigating the role that distancing between patient organizations and academia (universities, through contact with scientists and researchers, were "rarely" or "never" a preferred source for building knowledge by 19% of the organizations) may play.

STUDY LIMITATIONS

While achieving a 64% response rate for the questionnaire, this pilot study's scope was confined to Portuguese cancer-related patient organizations, a small universe. Given the low number of organizations with prior involvement in clinical research, the descriptive analysis provides limited insights into the correlation between organizational characteristics and participation in clinical research. To be able to draw broader conclusions, this questionnaire should be applied to a broader scope, allowing to explore these correlations along with variations across countries and disease areas.

Our findings do not offer an understanding of the reasons behind communication gaps between patients and researchers, also it falls short in elucidating how these patient organizations initiate or become involved in clinical studies. Future research should focus on unraveling the mechanisms behind the initiation of partnerships between patients and researchers.

Acknowledging the absence of the researchers' perspective in this study, we emphasize the importance of including it, as future work, for a more thorough understanding of patient involvement in cancer research. This can be achieved by developing a mirrored questionnaire specifically designed for researchers.

CONCLUSIONS

This study provides the first in-depth characterization of Portuguese cancer-related patient organizations and their views, motivations, and experiences on patient involvement in cancer research. Most importantly, this study revealed that most Portuguese cancer-related patient organizations were never involved in research but show high interest to be involved in different stages of a clinical study to ensure that research results are more aligned with the real needs of patients. The motivation expressed by all organizations to participate in clinical research should be turned into greater and more meaningful involvement in practice, through the develop of advocacy campaigns and public policies on patient involvement in cancer-research, so that the cancer community can benefit from the outcomes of a truly patient-centered research.

For peer review only

In the future, this questionnaire can be applied to other disease and umbrella patient organizations, at national and/or international levels, since it is not specific to any disease or the local context, contributing to a broader understanding of their and their views, motivations and experiences on patient involvement in research.

CONTRIBUTORSHIP STATEMENT

CR, TM, and AV conceptualized the study. CR, SC, TM, RSM, ARP and AV contributed to study design. CR, TM, RSM, ARP and AV contributed to data collection and CR, SC, RSM and ARP contributed to data analysis. CR and SC contributed to manuscript preparation. CR, SC, TM and RSM reviewed, edited, and approved the final manuscript. ARP and AV reviewed and approved the final manuscript. CR is responsible for the overall content as the guarantor.

FUNDING STATEMENT

This research received no specific grant from any funding agency in the public, commercial or not-for-profit sectors.

COMPETING INTERESTS STATEMENT

The authors declare that they have no known competing financial interests or personal relationships that could have appeared to influence the work reported in this paper.

DATA AVAILABILITY STATEMENT

All data relevant to the study are included in the article or uploaded as online supplemental information.

FIGURE LEGENDS

Figure 1. Survey development timeline.

Figure 2. Respondents' higher level of involvement in the 16 opportunities for patient involvement during the R&D life cycle proposed by Geissler *et al.* (2017).

Figure 3. Respondents' interest in participating and perceived level of influence in the 16 opportunities for patient involvement during the R&D life cycle proposed by Geissler *et al.* (2017).

For peer review only

REFERENCES

1. Advisory Committee of the People with AIDS. The Denver Principles [Internet]. 1983 [cited 2023 Jan 12]. Available from: https://data.unaids.org/pub/externaldocument/2007/gipa1983denverprinciples_en.pdf
2. Crimp D. Before Occupy: How AIDS Activists Seized Control of the FDA in 1988. The Atlantic [Internet]. [cited 2023 Jan 12]; Available from: https://www.theatlantic.com/health/archive/2011/12/before-occupy-how-aids-activists-seized-control-of-the-fda-in-1988/249302/
3. Lessem E. Nothing About Us Without Us: Community Engagement in Research to End the Epidemics. TAGline [Internet]. 2019 May [cited 2023 Jan 12]; Available from: https://www.treatmentactiongroup.org/wp-content/uploads/2011/08/tagline_may_2019_lessem_intro.pdf
4. Crisóstomo S, Alves da Costa F. Public Participation in Access to Medicines. In: Global Pharmaceutical Policy. Singapore: Springer Singapore; 2020. p. 261–91.
5. Charlton JI. Nothing About Us Without Us. University of California Press; 1998.
6. European Commission, Directorate-General for Research and Innovation. Special Eurobarometer 516 – April-May 2021: European citizens' knowledge and attitudes towards science and technology [Internet]. 2021. Available from: https://europa.eu/eurobarometer
7. Montgomery AA, Fahey T. How do patients' treatment preferences compare with those of clinicians? Qual Saf Health Care. 2001 Sep 1;10(Supplement 1):i39–43.
8. Sewitch MJ, Abrahamowicz M, Dobkin PL, Tamblyn R. Measuring Differences Between Patients' and Physicians' Health Perceptions: The Patient–Physician Discordance Scale. J Behav Med. 2003;26(3):245–64.
9. Harrison JD, Solomon MJ, Young JM, Meagher A, Butow P, Salkeld G, et al. Patient and Physician Preferences for Surgical and Adjuvant Treatment Options for Rectal Cancer. Archives of Surgery. 2008 Apr 1;143(4):389.
10. Report by the Secretariat of World Health Organization. Framework on integrated people-centred health services. [Internet]. 2016 [cited 2023 Jan 12]. Available from: http://apps.who.int/gb/ebwha/pdf_files/WHA69/A69_39-en.pdf?ua=1&ua=1
11. Deane K, Delbecque L, Gorbenko O, Hamoir AM, Hoos A, Nafria B, et al. Co-creation of patient engagement quality guidance for medicines development: an international multistakeholder initiative. BMJ Innov. 2019 Jan;5(1):43–55.
12. Fabbri A, Parker L, Colombo C, Mosconi P, Barbara G, Frattaruolo MP, et al. Industry funding of patient and health consumer organisations: systematic review with meta-analysis. BMJ. 2020 Jan 22;l6925.
13. Gentilini A, Miraldo M. The role of patient organisations in research and development: Evidence from rare diseases. Soc Sci Med. 2023 Dec 1;338.
14. Geissler J, Ryll B, di Priolo SL, Uhlenhopp M. Improving Patient Involvement in Medicines Research and Development: A Practical Roadmap. Ther Innov Regul Sci. 2017 Sep 30;51(5):612–9.
15. Warner K, See W, Haerry D, Klingmann I, Hunter A, May M. EUPATI Guidance for Patient Involvement in Medicines Research and Development (R&D); Guidance for Pharmaceutical Industry-Led Medicines R&D. Front Med (Lausanne). 2018 Oct 9;5.
16. Vat LE, Finlay T, Jan Schuitmaker-Warnaar T, Fahy N, Robinson P, Boudes M, et al. Evaluating the "return on patient engagement initiatives" in medicines research and development: A literature review. Health Expectations. 2020 Feb 6;23(1):5–18.

For peer review only

17. Cavaller-Bellaubi M, Faulkner SD, Teixeira B, Boudes M, Molero E, Brooke N, et al. Sustaining Meaningful Patient Engagement Across the Lifecycle of Medicines: A Roadmap for Action. Ther Innov Regul Sci. 2021 Sep 10;55(5):936–53.
18. Schandl A, Mälberg K, Haglund L, Arnberg L, Lagergren P. Patient and public involvement in oesophageal cancer survivorship research. Acta Oncol (Madr). 2022;61(3):371–7.
19. Souliotis K, Peppou LE, Agapidaki E, Tzavara C, Debiais D, Hasurdjiev S, et al. Health democracy in Europe: Cancer patient organization participation in health policy. Health Expectations. 2018 Apr 1;21(2):474–84.
20. Salamone JM, Lucas W, Brundage SB, Holloway JN, Stahl SM, Carbine NE, et al. Promoting scientist-advocate collaborations in cancer research: Why and how. Vol. 78, Cancer Research. American Association for Cancer Research Inc.; 2018. p. 5723–8.
21. Fagerlin A. Learning From Others Lessons for Improving Collaborations Between Stakeholders and Researchers [Internet]. Vol. 56. 2018. Available from: www.lww-medicalcare.com|S9
22. Gibson A, Welsman J, Britten N. Evaluating patient and public involvement in health research: from theoretical model to practical workshop. Health Expectations. 2017 Oct 1;20(5):826–35.
23. Brett J, Staniszewska S, Simera I, Seers K, Mockford C, Goodlad S, et al. Reaching consensus on reporting patient and public involvement (PPI) in research: Methods and lessons learned from the development of reporting guidelines. BMJ Open. 2017 Oct 1;7(10).
24. Schuster ALR, Hampel H, Paskett ED, Bridges JFP. Rethinking Patient Engagement in Cancer Research. Vol. 16, Patient. Adis; 2023. p. 89–93.
25. Gagnon MP, Tantchou Dipankui M, Poder TG, Payne-Gagnon J, Mbemba G, Beretta V. Patient and public involvement in health technology assessment: Update of a systematic review of international experiences. Int J Technol Assess Health Care. 2021;
26. Halvorsrud K, Kucharska J, Adlington K, Rüdell K, Brown Hajdukova E, Nazroo J, et al. Identifying evidence of effectiveness in the co-creation of research: a systematic review and meta-analysis of the international healthcare literature. Journal of Public Health (United Kingdom). 2021 Mar 1;43(1):197–208.
27. Pii KH, Schou LH, Piil K, Jarden M. Current trends in patient and public involvement in cancer research: A systematic review. Vol. 22, Health Expectations. Blackwell Publishing Ltd; 2019. p. 3–20.
28. Spears PA. Patient engagement in cancer research from the patient's perspective. Vol. 17, Future Oncology. Future Medicine Ltd.; 2021. p. 3717–28.
29. Faulkner SD, Pittens CACM, Goedhart NS, Davies EH, Manning E, Diaz-Ponce A, et al. Optimising Multi-stakeholder Practices in Patient Engagement: A Gap Analysis to Enable Focused Evolution of Patient Engagement in the Development and Lifecycle Management of Medicines. Ther Innov Regul Sci. 2021 Nov 1;55(6):1165–79.
30. Miller FA, Patton SJ, Dobrow M, Berta W. Public involvement in health research systems: A governance framework. Health Res Policy Syst. 2018 Aug 6;16(1).
31. Trio Presidency of the Council of the European Union, Germany, Portugal, Slovenia. Declaration on effective cancer research "Europe: Unite against Cancer" [Internet]. 2020 [cited 2023 Jan 12]. Available from: https://www.bmbf.de/SharedDocs/Downloads/en/210907-unite-against-cancer.pdf
32. German Federal Ministry of Education and Research. Principles of Successful Patient Involvement in Cancer Research [Internet]. 2021 [cited 2023 Jan 12]. Available from: https://bmbf.de/SharedDocs/Downloads/en/210907-unite-against-cancer.pdf
33. Amaro F, Pinto C, Carvalho MI. As Associações de Doentes em Portugal: Problemas, Necessidades e Aspirações. Abbvie, editor. Companhia das Ideias; 2015.

For peer review only

34. Bastos F. As associações de doentes em Portugal: profissionalização e estratégias de comunicação. [Internet]. ISCTE-IUL, Lisboa; 2018 [cited 2023 Jan 12]. Available from: https://repositorio.iscte-iul.pt/handle/10071/17677
35. Crisóstomo S, Santos M. Participação pública na saúde: das ideias à ação em Portugal. Rev Crit Cienc Sociais. 2018 Dec 1;(117):167–86.
36. de Freitas C. Public and patient participation in health policy, care and research. Porto Biomed J. 2017 Mar;2(2):31–2.
37. Corporate Consultation Secretariat, Health Policy and Communications Branch Minister of Public Works and Government Services Canada. Health Canada Policy Toolkit for Public Involvement in Decision Making [Internet]. 2000 [cited 2023 Jan 12]. Available from: https://www.canada.ca/content/dam/hc-sc/migration/hc-sc/ahc-asc/alt_formats/pacrb-dgapcr/pdf/public-consult/2000decision-eng.pdf
38. Landy DC, Brinich MA, Colten ME, Horn EJ, Terry SF, Sharp RR. How disease advocacy organizations participate in clinical research: a survey of genetic organizations. Genetics in Medicine. 2012 Feb;14(2):223–8.

For peer review only

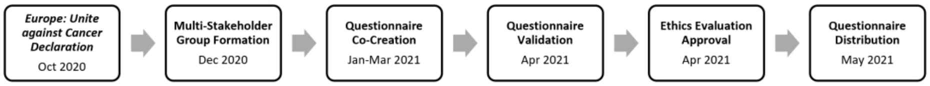

Figure 1. Survey development timeline.

480x123mm (130 x 130 DPI)

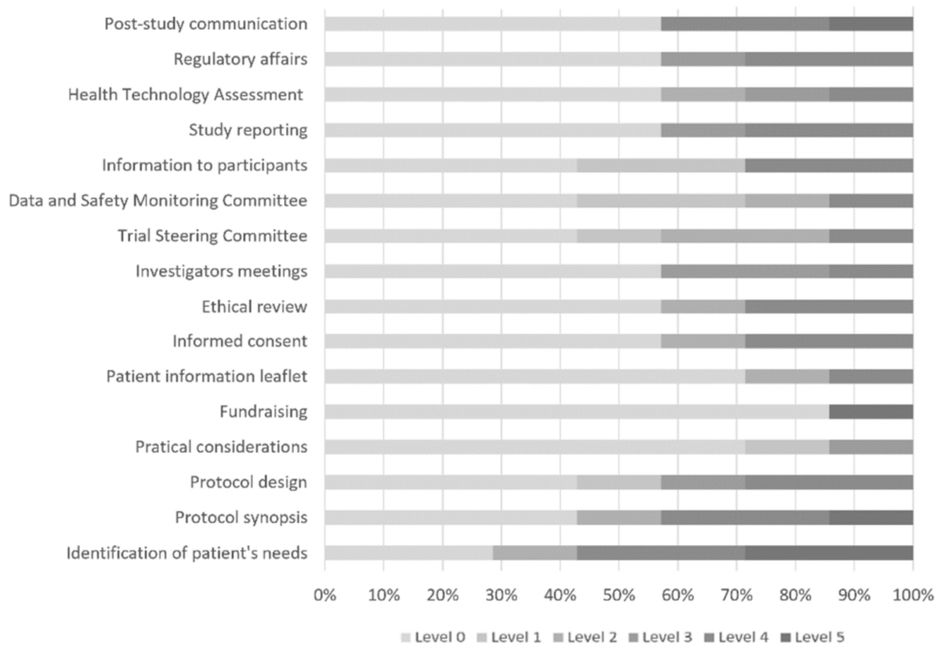

Figure 2. Respondents' higher level of involvement in the 16 opportunities for patient involvement during the R&D life cycle proposed by Geissler et al. (2017).

420x286mm (94 x 94 DPI)

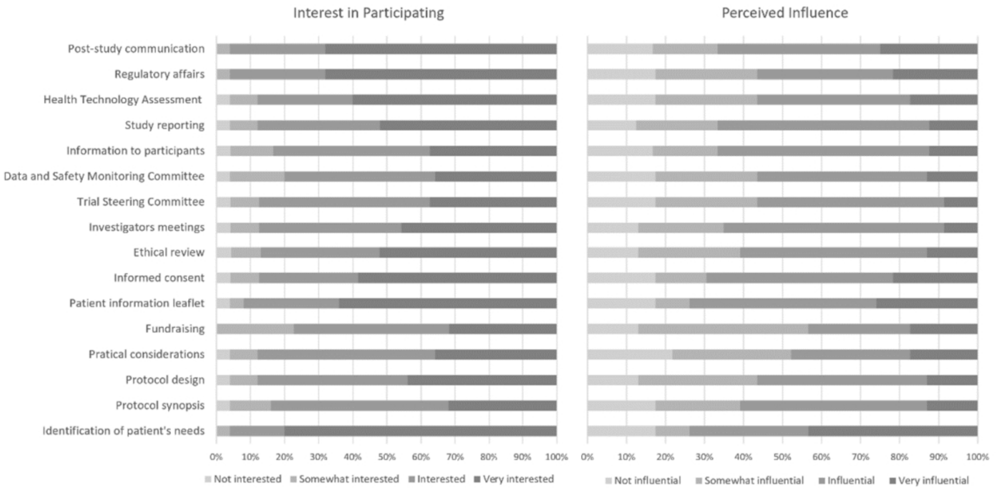

Figure 3. Respondents' interest in participating and perceived level of influence in the 16 opportunities for patient involvement during the R&D life cycle proposed by Geissler et al. (2017).

416x212mm (94 x 94 DPI)

NOVA SCHOOL OF BUSINESS & ECONOMICS

English (United Kingdom)

Introduction

Dear Sir/Madam,

This study is part of the research project “**Patient Involvement in Research**”, developed by a group of researchers and patient advocates, coordinated by Constança Roquette from the Nova School of Business and Economics, NOVA University of Lisbon. In the context of the Portuguese Presidency of the EU Council, and in response to the challenge posed by the *Europe: Unite against Cancer* declaration, this project aims to contribute to the understanding of **how patients and the organizations that represent them have been involved in research projects in the area of cancer, in Portugal.**

Therefore, a **questionnaire was developed for representatives of cancer patient organizations.** Through the responses obtained, it will be possible to analyze how organizations participate and are involved in research, as well as their perceptions, motivations and aspirations in this area.

To do this, we ask you to complete this questionnaire with 41 questions and an aproximate time of **30 minutes.**

Your participation in this study is **voluntary** and the non-participation will not cause you to be penalised. You can drop out of the study at any time.

Moreover, **the data from each organization will be encrypted** so their identity will not be assessed during data analysis. The **data will be clustered for analysis, rather than assessing the performance of each organization separately.** Only the Principal Investigator will have access to the encryption key, with the sole purpose of monitoring the number of responses.

This study received the approval of the Ethics Committee of the NOVA School of Business and Economics and is in full compliance with the General Data Protection Regulation (GDPR).**In case of any doubt, you can contact us** via email constanca.roquette@novasbe.pt.

From the results obtained with this questionnaire, **a space for joint and constructive reflection on the different opportunities for the involvement of patients in research will be created.** These will also be disclosed at a **meeting, to be held by the end of the Portuguese Presidency of the EU Council, in the 2nd quarter of this year**, where it is the intention to identify good practices, as well as discuss points of improvement for a more effective investigation that is closer to the real needs of patients.

If you want to be informed of the results and wish to be present at this meeting, fill out the registration form here.

We are very grateful for your participation.

I agree to participate in this study and confirm that I was informed about its conditions and that I have no questions in regard to it.

- ○ Yes.
- ○ No.

I answer this questionnaire on behalf of the following cancer patient organization:
(This information will be encrypted so the organization's identity will not be assessed during data analysis. The data will be clustered for analysis, rather than assessing the performance of each organization separately.)

[ ]

Do you identify yourself as the organization's usual representative?

- ○ Yes.

○ No. What is your role within the organization?

Clinical Research

Patients' participation in a clinical study *can occur at different stages, from participating in the design of the protocol, being present at meetings with researchers, participating in its dissemination during the recruitment phase to patients, disseminating results, among many others.*

Indicate the number and type of clinical studies your organization has been / is involved in.
(If you have not been involved in any of the types of studies presented, please mark with a "0" in the corresponding options.)

**Basic or pre-clinical studies** (laboratory studies to assess the biology of the disease or potential therapies and risks for humans. For example, pharmacological, pharmacokinetic and toxicological studies).	0
**Observational studies** (clinical studies without intervention, in which the participant is monitored by observation and data collection. It may, for example, be a study on the quality of life of people living with a certain disease / condition, by filling in a questionnaire).	0
**Clinical trials** (clinical studies with intervention, which may be any investigation conducted in humans, aimed at discovering or verifying the effects of one or more experimental drugs in order to determine their safety or efficacy).	0
**Others.** Which?	0
Total	0

What were the reasons that previously led to the non-participation of your organization in a clinical study?

- [ ] The organization was never asked to participate in this type of study.
- [ ] The organization was never able to respond to previous invitations to participate in a clinical study. Why?
- [ ] The organization does not consider its participation in a clinical study to be relevant.
- [ ] Other. Which?

Has your organization ever participated in another type of health research? (epidemiological, public health studies, etc.)

- ○ Yes.
- ○ No.

Who had the initiative to involve your organization in the clinical study(ies)?

- [ ] The initiative came from the organization itself.
  (through an associate or a Board member, for example.)
- [ ] The initiative came from others.
  (such as a research team, an individual investigator, or a patient, for example.)
- [ ] Other. Which?

Did the initiative to develop the study ever come from the organization itself?

- Yes.
- No.

Has the organization ever participated in the development of clinical recommendations?

- Yes.
- No.

Considering the clinical studies in which your organization was / is involved, select the option that represents the highest degree of involvement of your organization in each of the research stages.

Stage 1: Setting Research Priorities

At this stage, the organization:

	Did not participate or receive information (Level 0)	Has received information about this stage (Level 1)	Participated in information collection (Level 2)	Participated in the discussion of key points of the study (Level 3)	Participated in the discussion and actively decided together with the other partners (Level 4)	Participated as a full member of the research team, with equal decision-making power (Level 5)
Identification of the real needs of patients.	○	○	○	○	○	○

Stage 2: Research Design and Planning

At this stage, the organization:

	Did not participate or receive information (Level 0)	Has received information about this stage (Level 1)	Participated in information collection (Level 2)	Participated in the discussion of key points of the study (Level 3)	Participated in the discussion and actively decided together with the other partners (Level 4)	Participated as a full member of the research team, with equal decision-making power (Level 5)

	Did not participate or receive information (Level 0)	Has received information about this stage (Level 1)	Participated in information collection (Level 2)	Participated in the discussion of key points of the study (Level 3)	Participated in the discussion and actively decided together with the other partners (Level 4)	Participated as a full member of the research team, with equal decision-making power (Level 5)
**Study's synopsis design:** design and target population definition.	○	○	○	○	○	○
**Protocol design:** endpoints, inclusion and exclusion criteria, quality of life measures and patient-reported outcomes, crossover, ethical and data protection issues, recruitment and dissemination plan, retention measures, risk-benefit balance, etc.	○	○	○	○	○	○
**Consideration of practical aspects:** contractual issues, travel expenses and support for family members.	○	○	○	○	○	○
**Fundraising** for the research project.	○	○	○	○	○	○
**Patient Information sheet about the project:** content, visual design, readability, language and dissemination.	○	○	○	○	○	○
**Informed Consent:** content, visual design, readability and language.	○	○	○	○	○	○
**Ethical review** of the research project.	○	○	○	○	○	○

Stage 3: Research Conduct and Operations

At this stage, the organization:

	Did not participate or receive information (Level 0)	Has received information about this stage (Level 1)	Participated in information collection (Level 2)	Participated in the discussion of key points of the study (Level 3)	Participated in the discussion and actively decided together with the other partners (Level 4)	Participated as a full member of the research team, with equal decision-making power (Level 5)
**Investigators meeting to monitor the project:** representation of patients' perspectives on the study, recruitment, challenges, opportunities, which can trigger amendments.	○	○	○	○	○	○
**Trial Steering Committee:** protocol follow up, improvements in patient access and adherence to the study.	○	○	○	○	○	○
**Data and Safety Monitoring Committee:** risk / benefit assessment, drop-out issues and amendments to the project.	○	○	○	○	○	○

	Did not participate or receive information (Level 0)	Has received information about this stage (Level 1)	Participated in information collection (Level 2)	Participated in the discussion of key points of the study (Level 3)	Participated in the discussion and actively decided together with the other partners (Level 4)	Participated as a full member of the research team, with equal decision-making power (Level 5)
**Information to participants:** communication of protocol amendments and new safety information.	○	○	○	○	○	○
**Study reporting:** summary of interim results and dissemination in patient community.	○	○	○	○	○	○

Stage 4: Dissemination, Communication, Post-approval

At this stage, the organization:

	Did not participate or receive information (Level 0)	Has received information about this stage (Level 1)	Participated in information collection (Level 2)	Participated in the discussion of key points of the study (Level 3)	Participated in the discussion and actively decided together with the other partners (Level 4)	Participated as a full member of the research team, with equal decision-making power (Level 5)
**Health Technology Assessment:** assessment of value, patient-relevant outcomes and patient priorities.	○	○	○	○	○	○
**Regulatory affairs:** preparation of lay summaries of results, information leaflets and updated safety communications.	○	○	○	○	○	○
**Post-study communication:** contribution to publications, dissemination of research results to patient community / healthcare professionals.	○	○	○	○	○	○

What is your degree of agreement with the following statements regarding the overall participation of the organization in the clinical study(ies) in which it was / is involved?

	1. Strongly disagree.	2. Disagree.	3. Agree.	4. Strongly agree.	Rather not answer.
My organization's participation was valued by the research team in the course of the study.	○	○	○	○	○

	1. Strongly disagree.	2. Disagree.	3. Agree.	4. Strongly agree.	Rather not answer.
The contribution made by my organization was integrated / incorporated into the study.	○	○	○	○	○
My organization actively participated in the study.	○	○	○	○	○
There was an effective dialogue between my organization and the research team throughout the study.	○	○	○	○	○
My organization was recognized as a partner of equal relevance / weight in the study.	○	○	○	○	○
After participating in the study, the results were shared with my organization before being publicly disclosed.	○	○	○	○	○

Select the expressions that best represent the overall impact of your organization's participation in the clinical study(ies) in which it has been / is involved. (Select up to 3 expressions.)

- ☐ Increase in the relevance of the research.
- ☐ Increase in patient participation rates.
- ☐ Increase in the quality of the generated knowledged.
- ☐ Help in the better understanding of informed consent by the patients involved.
- ☐ Reduce in time for research completion.
- ☐ Reduce in financial costs of research.
- ☐ Minimize research participants risk.
- ☐ Others. Which?
- ☐ The impact of participation in the studies in which the organization was / is involved is unknown.

What main motivations do you identify in your organization to participate in clinical studies focused on the disease(s) to which it is dedicated? (Select up to 5 motivations.)

- ☐ Ensure that the study and its results address the real needs of patients.
- ☐ Gain research knowledge and skills.
- ☐ Increase the self-esteem and confidence of the patient(s) who represent the organization in the research process.
- ☐ Apply the experience and knowledge of patients about their condition.
- ☐ Promote the acceptance of patients as partners in a clinical study and increase the sense of co-creation of research.
- ☐ Increase access to finance that supports topics that would not otherwise be considered on the research agenda.
- ☐ Increase understanding of the nature and purpose of a clinical study.
- ☐ Improve understanding between patients and researchers.
- ☐ Develop health care and therapies that are more representative of patients' real needs.
- ☐ Promote data and information exchange between users and industry on the real use and management of a new drug / therapy (phase IV, pharmacovigilance).
- ☐ Others. Which?
- ☐ None of the above.

What is the interest of your organization in participating in the different stages of research* of the disease(s) to which it is dedicated?

	1. Not interested.	2. Somewhat interested.	3. Interested.	4. Very interested.	Rather not answer.
**1. Setting Research Priorities**					
Identification of the real needs of patients.	○	○	○	○	○
**2. Research Design and Planning**					
**Study's synopsis design:** design and target population definition.	○	○	○	○	○
**Protocol design:** endpoints, inclusion and exclusion criteria, quality of life measures and patient-reported outcomes, crossover, ethical and data protection issues, recruitment and dissemination plan, retention measures, risk-benefit balance, etc.	○	○	○	○	○

	1. Not interested.	2. Somewhat interested.	3. Interested.	4. Very interested.	Rather not answer.
**Consideration of practical aspects:** contractual issues, travel expenses and support for family members.	○	○	○	○	○
**Fundraising** for the research project.	○	○	○	○	○
**Patient Information sheet about the project:** content, visual design, readability, language and dissemination.	○	○	○	○	○
**Informed Consent:** content, visual design, readability and language.	○	○	○	○	○
**Ethical review** of the research project.	○	○	○	○	○
**3. Research Conduct and Operations**					
**Investigators meeting to monitor the project:** representation of patients' perspectives on the study, recruitment, challenges, opportunities, which can trigger amendments.	○	○	○	○	○
**Trial Steering Committee:** protocol follow up, improvements in patient access and adherence to the study.	○	○	○	○	○
**Data and Safety Monitoring Committee:** risk / benefit assessment, drop-out issues and amendments to the project.	○	○	○	○	○
**Information to participants:** communication of protocol amendments and new safety information.	○	○	○	○	○
**Study reporting:** summary of interim results and dissemination in patient community.	○	○	○	○	○
**4. Dissemination, Communication, Post-approval**					
**Health Technology Assessment:** assessment of value, patient-relevant outcomes and patient priorities.	○	○	○	○	○
**Regulatory affairs:** preparation of lay summaries of results, information leaflets and updated safety communications.	○	○	○	○	○
**Post-study communication:** contribution to publications, dissemination of research results to patient community / healthcare professionals.	○	○	○	○	○

What do you consider to be the influence of your organization in the different stages of research* of the disease(s) to which it is dedicated?

	1. Not influential.	2. Somewhat influential.	3. Influential.	4. Very influential.	Rather not answer.

	1. Not influential.	2. Somewhat influential.	3. Influential.	4. Very influential.	Rather not answer.
**1. Setting Research Priorities**					
Identification of the real needs of patients.	○	○	○	○	○
**2. Research Design and Planning**					
**Study's synopsis design:** design and target population definition.	○	○	○	○	○
**Protocol design:** endpoints, inclusion and exclusion criteria, quality of life measures and patient-reported outcomes, crossover, ethical and data protection issues, recruitment and dissemination plan, retention measures, risk-benefit balance, etc.	○	○	○	○	○
**Consideration of practical aspects:** contractual issues, travel expenses and support for family members.	○	○	○	○	○
**Fundraising** for the research project.	○	○	○	○	○
**Patient Information sheet about the project:** content, visual design, readability, language and dissemination.	○	○	○	○	○
**Informed Consent:** content, visual design, readability and language.	○	○	○	○	○
**Ethical review** of the research project.	○	○	○	○	○
**3. Research Conduct and Operations**					
**Investigators meeting to monitor the project:** representation of patients' perspectives on the study, recruitment, challenges, opportunities, which can trigger amendments.	○	○	○	○	○
**Trial Steering Committee:** protocol follow up, improvements in patient access and adherence to the study.	○	○	○	○	○
**Data and Safety Monitoring Committee:** risk / benefit assessment, drop-out issues and amendments to the project.	○	○	○	○	○
**Information to participants:** communication of protocol amendments and new safety information.	○	○	○	○	○
**Study reporting:** summary of interim results and dissemination in patient community.	○	○	○	○	○
**4. Dissemination, Communication, Post-approval**					
**Health Technology Assessment:** assessment of value, patient-relevant outcomes and patient priorities.	○	○	○	○	○
**Regulatory affairs:** preparation of lay summaries of results, information leaflets and updated safety communications.	○	○	○	○	○

	1. Not influential.	2. Somewhat influential.	3. Influential.	4. Very influential.	Rather not answer.
**Post-study communication:** contribution to publications, dissemination of research results to patient community / healthcare professionals.	◯	◯	◯	◯	◯

What is your perception of the influence that the following factors may have on the involvement of patient organizations in clinical studies?

	1. Not influential.	2. Somewhat influential.	3. Influential.	4. Very influential.
Participation in national and international collaborative networks.	◯	◯	◯	◯
Participation in medical and scientific events.	◯	◯	◯	◯
Visibility of the organization among the different stakeholders in the area of the disease on which it focuses.	◯	◯	◯	◯
Search for current and evidence-based information.	◯	◯	◯	◯
Existence of a collaborative culture / experience exchange between organizations and public institutions.	◯	◯	◯	◯
Existence of bureaucratic requirements / administrative procedures for the health system.	◯	◯	◯	◯
Researchers / Clinitians believe that patients are not able to participate in research.	◯	◯	◯	◯
Organizations own belief that patients are not able to participate in research	◯	◯	◯	◯
Researchers' lack of knowledge about how to involve patients in research.	◯	◯	◯	◯
Recognition of the importance of involving patients in research by the different stakeholders in the area of the disease.	◯	◯	◯	◯
Use of accessible and clear language for patients during research.	◯	◯	◯	◯
Interventional / participatory profile of the organization.	◯	◯	◯	◯
Knowledge of society in general on the subject of clinical research.	◯	◯	◯	◯
Knowledge of patients and organizations about the research process, as a whole, and about its different stages, in particular.	◯	◯	◯	◯

As initially mentioned, the aim of this study is to create a **space for joint and constructive reflection on the different opportunities for patients to be involved in research.**

Therefore, a **meeting will take place, by the end of the Portuguese Presidency of the Council of the EU, in the 2nd quarter of this year**, where it is the intention to identify good practices, as well as to discuss points of improvement for more effective research that is closer to the real needs of the patients.

In order to better prepare this meeting, we would like to ask you some questions.

How do you think your organization would like to be more involved in clinical research? (areas, projects or research phases, collaborations, etc.)

What clinical research topics would you like to discuss with other stakeholders in this area?

In what areas do you think that greater capacity is needed for patient organizations for better participation and more effective clinical research?

What would you like to say about your experience with bad practices in involving patients in research? What major problems can you identify?

Organization's Characteristics

This second part of the questionnaire aims to characterize your organization.

Organization Type

- ☐ Association.
- ☐ Foundation.
- ☐ Associative movement or other informal organization.
- ☐ Estatuto de Utilidade Pública. ****
- ☐ IPSS. ***
- ☐ NPO

Portuguese context..

Does the organization have a Scientific Advisory Board?

- ○ Yes.
- ○ No.

Do the organization's governing bodies include people with disease they represent?

	Yes	No
Board of Directors	○	○
Other governing bodies	○	○

Type of disease(s) to which the organization focuces on.

- ☐ Rare disease(s).
- ☐ Non rare disease(s).
- ☐ Single pathology.
- ☐ Multiple pathology.

Organization's foundation year.

[ ]

Annual budget *(based on the average value for the last 3 years, 2020 may be excluded if it is not representative, due to the Covid-19 pandemic).*

- ○ Up to 100 thousand euros.
- ○ 100 thousand to 250 thousand euros.
- ○ 250 thousand to 500 thousand euros.
- ○ 500 thousand to 1 million euros.
- ○ 1 million euros or more.

Number of associate members.

- ○ Less than 100 associate members.
- ○ 100 to 499 associate members.
- ○ 500 to 2999 associate members.
- ○ 3000 or more associate members.

Number of regular employees (estimate).

	0	1-3	4-10	11-30	> 30
Number of volunteer employees.	○	○	○	○	○
Number of paid employees.	○	○	○	○	○

Activities that best represent the organization's work.

- ☐ Development of educational materials.
- ☐ Organization of support groups.
- ☐ Organization of events for the patient education and other audiences.
- ☐ Financing of events for healthcare professionals.
- ☐ Financial assistance to patients.
- ☐ Advocacy / activism at the political level.
- ☐ Participation in conferences.
- ☐ Publications.
- ☐ Training for entities that are external to the organization.
- ☐ Collaboration in research projects.

☐ Others. Which?

Preferred sources of consultation for building knowledge / thinking and the frequency with which you consult them.

	1. Never.	2. Rarely.	3. Often.	4. Always.	Rather not answer.
Websites of national government institutions.	○	○	○	○	○
Websites of international organizations (World Health Organization, European Center for Disease Prevention and Control, etc.).	○	○	○	○	○
Scientific publications.	○	○	○	○	○
News in newspapers or magazines.	○	○	○	○	○
TV News.	○	○	○	○	○
Free search on the Internet (Google, Youtube, Podcasts).	○	○	○	○	○
Radio.	○	○	○	○	○
Participation in conferences / workshops / seminars.	○	○	○	○	○
Universities through contact with scientists and researchers.	○	○	○	○	○
Private companies (pharmaceutical, food laboratories, etc.)	○	○	○	○	○
Friends and / or family.	○	○	○	○	○
Healthcare professionals.	○	○	○	○	○

Thank you very much for your time and availability to answer these questions.

We would like to know a little more about your organization and, for that, we have 8 more questions that take about 5 minutes to answer.

Are you willing to continue a little further?

- ◯ Yes.
- ◯ No.

Geographic scope.

- ☐ National.
- ☐ Regional.
- ☐ Local.
- ☐ International.

Main sources of funding *(select the organization's top three sources of funding).*

- ☐ Membership fees from associates.
- ☐ Individual donations / fund-raising campaign.
- ☐ Management of the organization's equity or own funds.
- ☐ Services provision.
- ☐ European / International financing.
- ☐ Financing of private organizations (pharmaceutical industry, private companies, etc.)
- ☐ Public financing (Ministry of Health, Social Security, etc.)
- ☐ Personal income tax assignment.
- ☐ Others.

Associate members profile *(please also indicate the estimated percentage of members by type of situation that best characterizes them).*

Patients.	0	%
Relatives.	0	%

Healthcare professionals.	0	%
Others.	0	%
Total	0	%

Services available to associate members / community.

- ☐ Treatments.
- ☐ Internment.
- ☐ Clinical appointments.
- ☐ Nutrition consultations.
- ☐ Examinations / clinical analysis / screenings.
- ☐ Information about the disease.
- ☐ Psychological support.
- ☐ Assistance support.
- ☐ Economic and financial support.
- ☐ Information about legal rights.
- ☐ Residential support.
- ☐ Protocols for reimbursement and discounts (drugs, etc).
- ☐ Rental and availability of spaces.
- ☐ Services provided to the community (catering, laundry,....)
- ☐ Others. Which?

Communication channels with associate members and the community.

	Associate members	Community	Not used
Website / blog.	☐	☐	☐
Newsletter / bulletin / newspaper.	☐	☐	☐
Flyers / brochures.	☐	☐	☐
Email.	☐	☐	☐

	Associate members	Community	Not used
Telephone.	☐	☐	☐
Twitter.	☐	☐	☐
Facebook.	☐	☐	☐
Instagram.	☐	☐	☐
Linkedin.	☐	☐	☐
Youtube.	☐	☐	☐
Whatsapp.	☐	☐	☐

Does the organization have the following departments?

	Yes - Internal Department.	Yes - Outsourced.	No.
Marketing Department.	☐	☐	☐
Communications Department.	☐	☐	☐
Department of Public Relations.	☐	☐	☐

Does your organization participate in any National / European / International collaborative network?

	Yes.	No.
National	○	○
European / International	○	○

What collaborations are most relevant to the organization's day-to-day activities? (select until 5 collborations)

- ☐ Other patient organizations.
- ☐ National Directorate-General for Health
- ☐ Hospital Healthcare
- ☐ Private Sector / Companies / Pharmaceutical Laboratories
- ☐ Academia / Research Centers / Education Institutions

- ☐ Administrações Regionais de Saúde (ARS)
- ☐ Serviços Partilhados do Ministério da Saúde (SPMS)
- ☐ Academia / Centros de Investigação / Instituições de Educação.
- ☐ Sector Social / Fundações / Organizações sem fins lucrativos
- ☐ Sector Privado / Empresas / Laboratórios Farmacêuticos
- ☐ Cuidados de Saúde Primários.
- ☐ Cuidados de Saúde Hospitalares.
- ☐ Comissão de Ética para a Saúde (CES) externa.
- ☐ Instituto Nacional de Saúde Doutor Ricardo Jorge (INSA).
- ☐ Outra(s). Qual(ais)?

Portuguese context...

End

Thank you very much for your time and availability to answer this questionnaire!

As initially mentioned, the aim of this project is to create a **space for joint and constructive reflection on the different opportunities for patients to be involved in research.** The results of this questionnaire will be disclosed at a **meeting, to be held by the end of the Presidency, in the 2nd quarter of this year**, where it is the intention to identify good practices, as well as to discuss points of improvement for a more effective investigation that is closer to the real needs of patients.

If you would like to be informed of the results and wish to be present at this meeting, fill out the registration form here.

Powered by Qualtrics

NOVA SCHOOL OF BUSINESS & ECONOMICS

Português

Introdução

Exmo.(a) Sr.(a),

Este estudo insere-se no projeto de investigação "**Envolvimento de Doentes em Investigação**", desenvolvido por um grupo de investigadores e representantes das pessoas que vivem com doença, coordenado por Constança Roquette da NOVA School of Business and Economics. No contexto da Presidência Portuguesa do Conselho da UE, e em resposta ao desafio colocado pela declaração *Europe: Unite against Cancer*, este projeto pretende contribuir para o entendimento da forma **como doentes e as organizações que os representam têm vindo a ser envolvidos em projetos de investigação clínica, na área do cancro, em Portugal.**

Deste modo, foi desenvolvido um **questionário dirigido a representantes de organizações de doentes oncológicos.** Através das respostas obtidas, será possível analisar como as organizações participam e são envolvidas em investigação, bem como as suas percepções, motivações e aspirações nesta área.

Para tal, pedimos-lhe que preencha este questionário, com **41 perguntas e tempo aproximado de 30 minutos.**

A sua participação neste estudo é **voluntária** e a não participação não lhe trará qualquer prejuízo. Poderá desistir de participar neste estudo a qualquer momento.

A informação recolhida destina-se unicamente a fins de investigação e será somente tratada pelos investigadores. **Os dados serão tratados de forma codificada**, de modo a que a identidade da organização não seja associada às suas respostas. Os **dados serão tratados de forma agrupada, sem nunca ser analisada cada organização, em particular**. Apenas terá acesso à chave de codificação a Investigadora Principal do estudo, com o intuito único de

monitorização do número de respostas.

Este estudo mereceu o parecer favorável da Comissão de Ética da NOVA School of Business and Economics e está em total conformidade com o Regulamento Geral da Proteção de Dados (RGPD). **Em caso de dúvida poderá contactar-nos** através do e-mail constanca.roquette@novasbe.pt.

A partir dos resultados obtidos com este questionário, será criado um **espaço de reflexão conjunta e construtiva sobre as diferentes oportunidades de envolvimento de doentes em investigação.** Estes serão, também, divulgados num **encontro, a realizar até ao final da Presidência Portuguesa do Conselho da UE, no 2º trimestre deste ano**, onde se pretenderá identificar boas práticas, bem como discutir pontos de melhoria para uma investigação mais eficaz e mais próxima das necessidades reais dos doentes.

Caso pretenda ser informado dos resultados e deseje estar presente neste encontro, preencha o formulário de inscrição aqui.

Agradecemos a sua participação.

Aceito participar neste estudo, confirmando que fui esclarecido(a) sobre as condições do mesmo e que não tenho dúvidas.

- ○ Sim.
- ○ Não.

Respondo a este questionário em representação da seguinte organização de doentes oncológicos:

(Esta informação será codificada, de modo a não ser associada às suas respostas. Os dados serão tratados de forma agrupada, sem nunca ser analisada cada organização, em particular.)

Identifica-se como o representante habitual da organização?

- ○ Sim.

○ Não. Qual a sua função dentro da organização?

Investigação Clínica

*A **participação de doentes num estudo clínico** pode ocorrer em diferentes etapas do mesmo, desde participar na concepção do protocolo, estar presente em reuniões com investigadores, participar na sua divulgação durante a fase de recrutamento a doentes, disseminar resultados, entre muitas outras.*

Indique o número e tipo de estudos clínicos em que a sua organização esteve/está envolvida.

(Em caso de não ter estado envolvida em algum, ou nenhum, dos tipos de estudos apresentados, assinale, por favor, com um "0" nas opções correspondentes.)

**Estudos básicos ou pré-clínicos** *(estudos laboratoriais para avaliação da biologia da doença ou potenciais terapias e riscos para o ser humano. Por exemplo, estudos farmacológicos, farmacocinéticos e toxicológicos).*	0
**Estudos observacionais** *(estudos clínicos sem intervenção, em que é feito o acompanhamento do participante por observação e recolha de dados. Poderá, por exemplo, ser um estudo sobre a qualidade de vida das pessoas que vivem com determinada doença/condição, feito através do preenchimento de um questionário).*	0
**Ensaios clínicos** *(estudos clínicos com intervenção, podendo ser qualquer investigação conduzida no ser humano, destinada a descobrir ou a verificar os efeitos de um ou mais medicamentos experimentais a fim de apurar a respetiva segurança ou eficácia).*	0
**Outros.** Quais?	0
Total	0

Quais os motivos que levaram, anteriormente, à não participação da sua organização num estudo clínico?

- ☐ A organização nunca foi solicitada para participar neste tipo de estudo.
- ☐ A organização não teve possibilidade para dar resposta a anteriores convites para participar num estudo clínico. Porquê?
- ☐ A organização não considera relevante a sua participação num estudo clínico.
- ☐ Outra. Qual?

A sua organização alguma vez participou num outro tipo de investigação em saúde? (estudos epidemiológicos, de saúde pública, etc.)

- ○ Sim.
- ○ Não.

De quem partiu a iniciativa de envolver a sua organização no(s) estudo(s) clínico(s)?

- ☐ A iniciativa partiu da própria organização.
  (através de um associado ou de um membro Direcção, por exemplo.)
- ☐ A iniciativa partiu de outros.
  (como uma equipa de investigação, um investigador a título individual ou um doente, por exemplo.)
- ☐ Outro. Qual?

Em alguma das vezes, a iniciativa de desenvolver o estudo partiu da própria organização?

- Sim.
- Não.

A organização participou alguma vez na elaboração de recomendações clínicas?

- Sim.
- Não.

Considerando os estudos clínicos em que a sua organização esteve/está envolvida, assinale a opção que represente o maior grau de envolvimento da sua organização em cada uma das etapas de investigação.

Etapa 1: Definição das prioridades de investigação.

Nesta etapa, a organização:

	Não participou nem recebeu informações (Nível 0)	Recebeu informações sobre esta etapa (Nível 1)	Participou na recolha de informações (Nível 2)	Participou na discussão de pontos-chave (Nível 3)	Participou na discussão e decidiu ativamente, em conjunto com os outros parceiros (Nível 4)	Participou como membro *de facto* da equipa de investigação, com igual poder de decisão (Nível 5)
Identificação das necessidades reais dos doentes.	○	○	○	○	○	○

Etapa 2: Desenho e planeamento do projeto de investigação

Nesta etapa, a organização:

	Não participou nem recebeu informações (Nível 0)	Recebeu informações sobre esta etapa (Nível 1)	Participou na recolha de informações (Nível 2)	Participou na discussão de pontos-chave (Nível 3)	Participou na discussão e decidiu ativamente, em conjunto com os outros parceiros (Nível 4)	Participou como membro *de facto* da equipa de investigação, com igual poder de decisão (Nível 5)
**Elaboração da sinopse / sumário do estudo:** desenho e definição da população alvo.	◯	◯	◯	◯	◯	◯
**Elaboração do protocolo:** definição de parâmetros relevantes, critérios de inclusão e exclusão, medidas de qualidade de vida e resultados reportados pelos doentes (*patient-reported outcomes*), desenho do estudo, questões de ética e de proteção de dados, plano de recrutamento e disseminação do estudo, medidas de retenção, análise risco-benefício, etc.	◯	◯	◯	◯	◯	◯
**Consideração de aspectos práticos:** questões contratuais, despesas de viagem/deslocação e apoio a familiares.	◯	◯	◯	◯	◯	◯
**Angariação de fundos** para o projeto de investigação.	◯	◯	◯	◯	◯	◯
**Folha de Informação aos doentes sobre o projeto:** conteúdo, design visual, legibilidade, linguagem e disseminação.	◯	◯	◯	◯	◯	◯
**Consentimento Informado**: conteúdo, *design* visual, legibilidade e linguagem.	◯	◯	◯	◯	◯	◯
**Revisão ética** do projeto de investigação.	◯	◯	◯	◯	◯	◯

Etapa 3: Condução da investigação e processos associados

Nesta etapa, a organização:

	Não participou nem recebeu informações (Nível 0)	Recebeu informações sobre esta etapa (Nível 1)	Participou na recolha de informações (Nível 2)	Participou na discussão de pontos-chave (Nível 3)	Participou na discussão e decidiu ativamente, em conjunto com os outros parceiros (Nível 4)	Participou como membro *de facto* da equipa de investigação, com igual poder de decisão (Nível 5)
**Reunião de Investigadores para acompanhamento do projeto:** representação das perspetivas dos doentes quanto ao estudo, recrutamento, desafios, oportunidades, podendo originar emendas.	◯	◯	◯	◯	◯	◯

	Não participou nem recebeu informações (Nível 0)	Recebeu informações sobre esta etapa (Nível 1)	Participou na recolha de informações (Nível 2)	Participou na discussão de pontos-chave (Nível 3)	Participou na discussão e decidiu ativamente, em conjunto com os outros parceiros (Nível 4)	Participou como membro *de facto* da equipa de investigação, com igual poder de decisão (Nível 5)
**Comissão de gestão / monitorização do estudo:** seguimento *(follow up)* do protocolo, melhorias no acesso e adesão dos doentes ao estudo.						
**Comissão de Monitorização da Segurança dos Dados**: avaliação de riscos/benefícios, motivos de desistências e emendas ao projeto.						
**Informação aos participantes:** comunicação de emendas do projeto e novas informações de segurança.						
**Relatório do estudo:** sumário de resultados provisórios, disseminação pela comunidade de doentes.						

Etapa 4: Disseminação, Comunicação e Atividades Pós-Aprovação.

Nesta etapa, a organização:

	Não participou nem recebeu informações (Nível 0)	Recebeu informações sobre esta etapa (Nível 1)	Participou na recolha de informações (Nível 2)	Participou na discussão de pontos-chave (Nível 3)	Participou na discussão e decidiu ativamente, em conjunto com os outros parceiros (Nível 4)	Participou como membro *de facto* da equipa de investigação, com igual poder de decisão (Nível 5)
**Avaliação de Tecnologias de Saúde (*Health Technology Assessment*):** avaliação de valor, relevância de resultados e prioridades para os doentes.						
**Assuntos regulamentares:** elaboração de resumos em linguagem leiga, folhetos informativos e comunicações de segurança atualizadas.						
**Comunicação após estudo:** contribuição para publicações, disseminação de resultados do estudo na comunidade de doentes e/ou profissionais de saúde.						

Qual o seu grau de concordância com as seguintes afirmações relativamente à globalidade da participação da organização no(s) estudo(s) clínico(s) em que esteve/está envolvida?

	1. Discordo fortemente.	2. Discordo.	3. Concordo.	4. Concordo fortemente.	Pı ı resı
A participação da minha organização foi valorizada pela equipa de investigação no decorrer do estudo.	◯	◯	◯	◯	
O contributo dado pela minha organização foi integrado/incorporado no estudo.	◯	◯	◯	◯	
A minha organização participou ativamente na investigação.	◯	◯	◯	◯	
Houve efetivo diálogo entre a minha organização e a equipa de investigadores ao longo do estudo.	◯	◯	◯	◯	
A minha organização foi reconhecida como parceira de igual relevância/peso no estudo.	◯	◯	◯	◯	
Após participação no estudo, os resultados foram partilhados com a minha organização antes de serem publicamente divulgados.	◯	◯	◯	◯	

Selecione as expressões que melhor representam o impacto global da participação da sua organização no(s) estudo(s) clínicos em que esteve/está envolvida. (Selecione até 3 expressões.)

- ☐ Aumento da relevância da investigação.
- ☐ Aumento da taxa de participação de doentes.
- ☐ Aumento da qualidade do conhecimento gerado.
- ☐ Ajuda na melhor compreensão do consentimento informado por parte dos doentes envolvidos.
- ☐ Diminuição do tempo necessário para completar o estudo.
- ☐ Diminuição dos custos do estudo.
- ☐ Minimização dos riscos de participação para os doentes.
- ☐ Outros. Quais?

☐ O impacto da participação nos estudos em que a organização esteve envolvida é desconhecido.

Que principais motivações identifica na sua organização para participar em estudos clínicos sobre a(s) doença(s) a que se dedica? (Selecione até 5 motivações.)

☐ Garantir que o estudo e os seus resultados abordam reais necessidades dos doentes.

☐ Ganhar conhecimento e competências de investigação.

☐ Aumentar a auto-estima e confiança do(s) doente(s) que representa(m) a organização no processo de investigação.

☐ Aplicar a experiência e conhecimento dos doentes sobre a sua condição.

☐ Promover a aceitação dos doentes como parceiros num estudo clínico e aumento de sentido de co-criação da investigação.

☐ Aumentar o acesso a financiamento que permita colocar na agenda de investigação tópicos que de outro modo não seriam considerados.

☐ Aumentar a compreensão da natureza e propósito de um estudo clínico.

☐ Melhorar o entendimento entre doentes e investigadores.

☐ Desenvolver cuidados de saúde e terapias que são mais representativas das reais necessidades dos doentes.

☐ Promover a troca de dados e informações entre utilizadores e indústria sobre a realidade do uso e gestão de um novo medicamento / terapia (fase IV, farmacovigilância).

☐ Outros. Quais?

☐ Nenhumas.

Qual o interesse da sua organização em participar nas diferentes etapas de investigação da(s) doença(s) a que se dedica?

	1. Nada interessada.	2. Pouco interessada.	3. Interessada.	4. Muito interessada.	Prefiro não responder.

	1. Nada interessada.	2. Pouco interessada.	3. Interessada.	4. Muito interessada.	Prefiro não responder.
**1. Definição das prioridades de investigação**					
Identificação das necessidades reais dos doentes.	○	○	○	○	○
**2. Desenho e planeamento do projeto de investigação**					
**Elaboração da sinopse / sumário do estudo:** desenho e definição da população alvo.	○	○	○	○	○
**Elaboração do protocolo:** definição de parâmetros relevantes, critérios de inclusão e exclusão, medidas de qualidade de vida e resultados reportados pelos doentes (*patient-reported outcomes*), desenho do estudo, questões de ética e de proteção de dados, plano de recrutamento e disseminação do estudo, medidas de retenção, análise risco-benefício, etc.	○	○	○	○	○
**Consideração de aspectos práticos:** questões contratuais, despesas de viagem/deslocação e apoio a familiares.	○	○	○	○	○
**Angariação de fundos** para o projeto de investigação.	○	○	○	○	○
**Folha de Informação aos doentes sobre o projeto:** conteúdo, design visual, legibilidade, linguagem e disseminação.	○	○	○	○	○
**Consentimento Informado**: conteúdo, *design* visual, legibilidade e linguagem.	○	○	○	○	○
**Revisão ética** do projeto de investigação.	○	○	○	○	○
**3. Condução da investigação e processos associados**					
**Reunião de Investigadores para acompanhamento do projeto:** representação das perspetivas dos doentes quanto ao estudo, recrutamento, desafios, oportunidades, podendo originar emendas.	○	○	○	○	○
**Comissão de gestão / monitorização do estudo:** seguimento *(follow up)* do protocolo, melhorias no acesso e adesão dos doentes ao estudo.	○	○	○	○	○
**Comissão de Monitorização da Segurança dos Dados**: avaliação de riscos/benefícios, motivos de desistências e emendas ao projeto.	○	○	○	○	○
**Informação aos participantes:** comunicação de emendas do projeto e novas informações de segurança.	○	○	○	○	○
**Relatório do estudo:** sumário de resultados provisórios, disseminação pela comunidade de doentes.	○	○	○	○	○

	1. Nada interessada.	2. Pouco interessada.	3. Interessada.	4. Muito interessada.	Prefiro não responder.
**4. Disseminação, Comunicação e Atividades Pós-Aprovação**					
**Avaliação de Tecnologias de Saúde (*Health Technology Assessment)*:** avaliação de valor, relevância de resultados e prioridades para os doentes.	○	○	○	○	○
**Assuntos regulamentares:** elaboração de resumos em linguagem leiga, folhetos informativos e comunicações de segurança atualizadas.	○	○	○	○	○
**Comunicação após estudo:** contribuição para publicações, disseminação de resultados do estudo na comunidade de doentes e/ou profissionais de saúde.	○	○	○	○	○

Qual considera ser a influência da sua organização nas diferentes etapas de investigação da(s) doença(s) a que se dedica?

	1. Nada influente.	2. Pouco influente.	3. Influente.	4. Muito influente.	Prefiro não responder.
**1. Definição das prioridades de investigação**					
Identificação das necessidades reais dos doentes.	○	○	○	○	○
**2. Desenho e planeamento do projeto de investigação**					
**Elaboração da sinopse / sumário do estudo:** desenho e definição da população alvo.	○	○	○	○	○
**Elaboração do protocolo:** definição de parâmetros relevantes, critérios de inclusão e exclusão, medidas de qualidade de vida e resultados reportados pelos doentes (*patient-reported outcomes*), desenho do estudo, questões de ética e de proteção de dados, plano de recrutamento e disseminação do estudo, medidas de retenção, análise risco-benefício, etc.	○	○	○	○	○
**Consideração de aspectos práticos:** questões contratuais, despesas de viagem/deslocação e apoio a familiares.	○	○	○	○	○
**Angariação de fundos** para o projeto de investigação.	○	○	○	○	○
**Folha de Informação aos doentes sobre o projeto:** conteúdo, design visual, legibilidade, linguagem e disseminação.	○	○	○	○	○

	1. Nada influente.	2. Pouco influente.	3. Influente.	4. Muito influente.	Prefiro não responder.
**Consentimento Informado**: conteúdo, *design* visual, legibilidade e linguagem.	○	○	○	○	○
**Revisão ética** do projeto de investigação.	○	○	○	○	○

3. Condução da investigação e processos associados

**Reunião de Investigadores para acompanhamento do projeto:** representação das perspetivas dos doentes quanto ao estudo, recrutamento, desafios, oportunidades, podendo originar emendas.	○	○	○	○	○
**Comissão de gestão / monitorização do estudo:** seguimento *(follow up)* do protocolo, melhorias no acesso e adesão dos doentes ao estudo.	○	○	○	○	○
**Comissão de Monitorização da Segurança dos Dados**: avaliação de riscos/benefícios, motivos de desistências e emendas ao projeto.	○	○	○	○	○
**Informação aos participantes:** comunicação de emendas do projeto e novas informações de segurança.	○	○	○	○	○
**Relatório do estudo:** sumário de resultados provisórios, disseminação pela comunidade de doentes.	○	○	○	○	○

4. Disseminação, Comunicação e Atividades Pós-Aprovação

**Avaliação de Tecnologias de Saúde (*Health Technology Assessment)*:** avaliação de valor, relevância de resultados e prioridades para os doentes.	○	○	○	○	○
**Assuntos regulamentares:** elaboração de resumos em linguagem leiga, folhetos informativos e comunicações de segurança atualizadas.	○	○	○	○	○
**Comunicação após estudo:** contribuição para publicações, disseminação de resultados do estudo na comunidade de doentes e/ou profissionais de saúde.	○	○	○	○	○

Qual a sua perceção sobre a influência, negativa ou positiva, que podem ter os seguintes fatores no envolvimento das organizações de doentes em estudos clínicos?

	1. Nada influente.	2. Pouco influente.	3. Influente.	4. Muito influente.	Prefiro não responder.
Participação em redes colaborativas nacionais e internacionais.	○	○	○	○	○
Participação em congressos e eventos da comunidade médica e científica.	○	○	○	○	○

	1. Nada influente.	2. Pouco influente.	3. Influente.	4. Muito influente.	Prefiro não responder.
Visibilidade da organização entre os diferentes atores da área da doença em que se foca.	○	○	○	○	○
Procura por informação atual e baseada em evidência.	○	○	○	○	○
Existência de cultura / experiência de colaboração entre associações e instituições públicas.	○	○	○	○	○
Existência de procedimentos / requisitos burocráticos complexos na inclusão de doentes em estudos	○	○	○	○	○
Convicção, por parte dos investigadores/médicos, de que os doentes não têm capacidade para participar em investigação.	○	○	○	○	○
Convicção, por parte das próprias organizações, de que os doentes não têm capacidade para participar em investigação.	○	○	○	○	○
Desconhecimento sobre como envolver os doentes em investigação por parte dos investigadores.	○	○	○	○	○
Reconhecimento, por parte dos diferentes atores da área de doença, da importância do envolvimento dos doentes em investigação.	○	○	○	○	○
Utilização de linguagem acessível e clara para os doentes no decorrer da investigação.	○	○	○	○	○
Carácter interventivo / participativo da organização.	○	○	○	○	○
Conhecimento da sociedade em geral sobre o tema da investigação clínica.	○	○	○	○	○
Conhecimento dos doentes e das organizações sobre o processo de investigação como um todo e sobre as diferentes etapas, em particular.	○	○	○	○	○

Como referido inicialmente, pretende-se com este estudo criar um **espaço de reflexão conjunta e construtiva sobre as diferentes oportunidades de envolvimento de doentes em investigação.**

Para tal, irá realizar-se um **encontro, até ao final da Presidência Portuguesa do Conselho da UE, no 2º trimestre deste ano**, onde se pretenderá identificar boas práticas, bem como

discutir pontos de melhoria para uma investigação mais eficaz e mais próxima das necessidades reais dos doentes.

Com o intuito de melhor preparar este momento, gostaríamos de lhe fazer algumas perguntas.

Como considera que a sua organização gostaria de estar mais envolvida na investigação clínica? (áreas, projetos ou fases de investigação, colaborações, etc.)

Que temas de investigação clínica gostaria de discutir com os demais intervenientes nesta área?

Em que áreas considera que é necessária maior capacitação das organizações de doentes para uma melhor participação e mais eficaz investigação clínica?

O que gostaria de referir quanto à sua experiência com más práticas no envolvimento de doentes em investigação? Que problemas de maior relevância consegue identificar?

Caracterização da Organização

Esta segunda parte do questionário destina-se à caracterização da sua organização.

Tipo de organização.

- [ ] Associação.

- ☐ Fundação.
- ☐ Movimento ou outra organização informal.
- ☐ Estatuto de Utilidade Pública.
- ☐ IPSS.
- ☐ ONGD.

A organização tem um Conselho Científico / Consultivo?

- ○ Sim.
- ○ Não.

Os órgãos sociais da organização incluem pessoas com a doença que representam?

	Sim	Não
Direção	○	○
Outros órgãos sociais	○	○

Tipo de doença(s) a que a organização se dedica.

- ☐ Doença(s) rara(s).
- ☐ Doença(s) não rara(s).
- ☐ Uma só patologia.
- ☐ Multi-patologia.

Ano de fundação da organização.

Orçamento anual *(tendo por referência o valor médio dos últimos 3 anos, podendo excluir-se o ano 2020, se considerar não representativo, devido à pandemia por Covid-19).*

- ○ Até 100 mil euros.

- ◯ 100 mil a 250 mil euros.
- ◯ 250 mil a 500 mil euros.
- ◯ 500 mil a 1 milhão de euros.
- ◯ 1 milhão de euros ou superior.

Número de associados.

- ◯ Menos de 100 associados.
- ◯ 100 a 499 associados.
- ◯ 500 a 2999 associados.
- ◯ 3000 ou mais associados.

Número de colaboradores regulares (estimativa).

	0	1-3	4-10	11-30	> 30
Número de colaboradores voluntários.	◯	◯	◯	◯	◯
Número de colaboradores remunerados.	◯	◯	◯	◯	◯

Atividades que melhor representam o trabalho da organização.

- ☐ Desenvolvimento de materiais educativos.
- ☐ Organização de grupos de apoio.
- ☐ Organização de eventos para educação de doentes e outros públicos.
- ☐ Financiamento de eventos de profissionais de saúde.
- ☐ Assistência financeira a doentes.
- ☐ Advocacy/ativismo a nível político.
- ☐ Participação em congressos.
- ☐ Publicações.
- ☐ Formação a entidades externas à organização.

- ☐ Colaboração em investigação.
- ☐ Outra(s). Qual(ais)?

Fontes preferenciais de consulta para construção de conhecimento / pensamento e a frequência com que as consulta.

	1. Nunca.	2. Com pouca frequência.	3. Com muita frequência.	4. Sempre.	re
Websites de instituições governamentais nacionais (Ministério da Saúde, Direção Geral de Saúde, etc.)	○	○	○	○	
Websites de organizações internacionais (Organização Mundial de Saúde, European Centre for Disease Prevention and Control, etc.)	○	○	○	○	
Publicações científicas.	○	○	○	○	
Notícias em jornais ou revistas.	○	○	○	○	
Telejornais.	○	○	○	○	
Pesquisa livre na Internet (Google, Youtube, Podcasts).	○	○	○	○	
Rádio.	○	○	○	○	
Participação em conferências/ workshops/ congressos.	○	○	○	○	
Universidades através do contacto com cientistas e investigadores.	○	○	○	○	
Empresas privadas (laboratórios farmacêuticos, alimentares, etc.)	○	○	○	○	
Amigos e / ou familiares.	○	○	○	○	
Profissionais de saúde.	○	○	○	○	

Muito obrigado pelo seu tempo e disponibilidade para responder a estas perguntas.

Gostaríamos de saber um pouco mais sobre a sua organização e, para tal, temos mais 8 perguntas que demoram cerca de 5 minutos a responder.

Tem disponibilidade para prosseguir um pouco mais?

- ○ Sim.
- ○ Não.

Âmbito geográfico.

- ☐ Nacional (Portugal).
- ☐ Regional.
- ☐ Local.
- ☐ Internacional.

Principais fontes de financiamento *(selecione as três principais fontes de financiamento da organização).*

- ☐ Quotas de Associados.
- ☐ Donativos Individuais / Peditório.
- ☐ Gestão de Património ou de Fundos Próprios da Organização.
- ☐ Prestação de Serviços.
- ☐ Financiamento Europeu / Internacional.
- ☐ Financiamento de Organizações Privadas (Indústria Farmacêutica, Empresas Privadas, etc.)
- ☐ Financiamento Público (Ministério da Saúde, Segurança Social, etc.)
- ☐ Consignação do IRS.
- ☐ Outros.

Perfil dos associados *(indique, também, por favor, a percentagem (estimada) de associados por tipo de situação que melhor os caracteriza).*

Pessoas com doença.	0	%
Familiares.	0	%
Profissionais de saúde.	0	%
Outros.	0	%
Total	0	%

Serviços disponibilizados aos associados/comunidade.

- [ ] Tratamentos.
- [ ] Internamento.
- [ ] Consultas clínicas.
- [ ] Consultas de nutrição.
- [ ] Exames / análises clínicas / rastreios.
- [ ] Informação sobre a doença.
- [ ] Apoio psicológico.
- [ ] Apoio assistencial.
- [ ] Apoio económico-financeiro.
- [ ] Informação sobre direitos.
- [ ] Apoio residencial.
- [ ] Protocolos para comparticipação e descontos (medicamentos, etc).
- [ ] Aluguer e disponibilização de espaços.
- [ ] Serviços prestados à comunidade (catering, lavandaria, ...)
- [ ] Outros? Quais?

Veículos de comunicação com associados e comunidade.

	Associados	Comunidade	Não usa
Página / blog na internet.	☐	☐	☐

	Associados	Comunidade	Não usa
Newsletter / boletim / jornal.	☐	☐	☐
Folhetos / brochuras.	☐	☐	☐
Email.	☐	☐	☐
Telefone.	☐	☐	☐
Twitter.	☐	☐	☐
Facebook.	☐	☐	☐
Instagram.	☐	☐	☐
Linkedin.	☐	☐	☐
Youtube.	☐	☐	☐
Whatsapp.	☐	☐	☐

A organização tem os seguintes departamentos?

	Sim - Departamento Interno.	Sim - Contratação Externa.	Não.
Departamento de Marketing.	☐	☐	☐
Departamento de Comunicação.	☐	☐	☐
Departamento de Relações Públicas.	☐	☐	☐

A sua organização participa em alguma rede ou organização Nacional / Europeia / Internacional?

	Sim.	Não.
Nacional	○	○
Europeia / Internacional	○	○

Que colaborações nacionais são mais relevantes para as atividades do dia-à-dia da sua organização? (selecione até 5 colaborações)

☐ Outras organizações de doentes.

☐ Ministério da Saúde.

- [ ] INFARMED, I.P..
- [ ] Direção-Geral de Saúde (DGS).
- [ ] Administração Central do Sistema de Saúde, I.P. (ACSS).
- [ ] Administrações Regionais de Saúde (ARS)
- [ ] Serviços Partilhados do Ministério da Saúde (SPMS)
- [ ] Academia / Centros de Investigação / Instituições de Educação.
- [ ] Sector Social / Fundações / Organizações sem fins lucrativos
- [ ] Sector Privado / Empresas / Laboratórios Farmacêuticos
- [ ] Cuidados de Saúde Primários.
- [ ] Cuidados de Saúde Hospitalares.
- [ ] Comissão de Ética para a Saúde (CES) externa.
- [ ] Instituto Nacional de Saúde Doutor Ricardo Jorge (INSA).
- [ ] Outra(s). Qual(ais)?

Fim

Muito obrigado pelo seu tempo e disponibilidade para responder a este questionário!

Como referido anteriormente, pretende-se com este estudo criar um **espaço de reflexão conjunta e construtiva sobre as diferentes oportunidades de envolvimento de doentes em investigação.** Os resultados deste questionário serão divulgados num **encontro, a realizar até ao final da Presidência, no 2º trimestre deste ano**, onde se pretenderá identificar boas práticas, bem como discutir pontos de melhoria para uma investigação mais eficaz e mais próxima das necessidades reais dos doentes.

Caso pretenda ser informado dos resultados e deseje estar presente neste encontro, preencha o formulário de inscrição aqui.

Partilhamos também algumas referências em que nos baseámos para construir este questionário.

[Etapas de Investigação e Oportunidades de Envolvimento de Doentes]

Geissler J et al. (2017), *Improving patient involvement in medicines research and development: a practical roadmap. Therapeutic Innovation and Regulatory Science*, 51:612–19.

[Níveis de Envolvimento]

Health Canada Policy Toolkit for Public Involvement in Decision Making (2000), Corporate Consultation Secretariat, Health Policy and Communications Branch Minister of Public Works and Government Services Canada.

[Estudo focado nas organizações de doentes, no contexto das doenças genéticas, de forma a avaliar a sua participação em investigação clínica]

Landy, D., Brinich, M., Colten, M. et al. (2012) *How disease advocacy organizations participate in clinical research: a survey of genetic organizations. Genet Med* 14, 223–228.

Powered by Qualtrics

view only

Checklist for Reporting Results of Internet E-Surveys (CHERRIES)

Item Category	*Checklist Item*	*Description*
**Design**		The survey was designed with Qualtrics^{TM} software, and the target sample was the universe of cancer-related patient organizations' representatives in Portugal. The final list comprised 42 cancer-related patient organizations that represent cancer patients, their caregivers and other affected and interested people, who often give wider voice to patients' needs.
**IRB**	**IRB approval**	The questionnaire received the approval of the Ethics Committee of the NOVA School of Business and Economics on April 7th, 2020.
	**Informed consent**	On the survey welcome page, the participants were informed about the objectives of the study, the target audience, and that the questionnaire had 41 questions and an approximate time of completion of 30 minutes. A consent request was presented at the end of the introduction and participants could only access the questionnaire if they agree with it.
	**Data protection**	The respondents' data were clustered for analysis, without assessing each organization's performance being assessed separately. The data from each organization was encrypted and therefore its identity was not accessed during data analysis. Only the Principal Investigator had access to the encryption key (automatically generated by the Qualtrics software), with the sole purpose of monitoring the responses and to confirm if the organizations reached were indeed the ones on the target sample.
**Development and testing**		A focus group of seven patient organization's representatives was organized to test the survey for content validity and comprehensiveness. Given the small size of the target sample, this focus group was constituted by non-cancer patient organization's representatives to ensure a similar context. A 3-hour session took place online, providing room for discussion and constructive feedback. The final version of the survey was finalized based on the feedback collected, which proved to be valuable for the instrument improvement.
**Recruitment process**	**Open survey versus closed survey**	The survey was an open survey, distributed to the 42 cancer-related patient organizations' representatives via email.
	**Contact mode**	The survey was sent by email and stayed active for 2 weeks, and all the organizations were contacted by phone as a follow-up to the initial email. After the first week, all non-responders received a reminder followed by a second phone call and, finally, two days before the end date, another email was sent.
	**Advertising the survey**	The survey was not advertised; it was sent directly by email to the 42 cancer-related patient organizations' representatives in Portugal.
**Survey Administration**	**Web/E-mail**	This was an online survey hosted by Qualtrics^{TM}. Participants accessed the survey by clicking on the link presented on the email.

For peer review only

	**Context**	On May 10th, 2020, the online survey designed with Qualtrics™ software was distributed to the 42 cancer-related patient organizations' representatives via email.
	**Mandatory/voluntary**	Participation was voluntary, and this was outlined to participants during the survey introduction before the informed consent request.
	**Incentives**	Participants were not financially compensated, but the working group made a commitment to share and discuss the findings publicly and with all participants who showed interest.
	**Time/Date**	Responses were collected between May 10th and May 24th, 2020.
	**Randomization of items or questionnaires**	No randomization of items was used.
	**Adaptive questioning**	Adaptive questioning was used. Relevant survey items were displayed or hidden based on previous items' responses.
	**Number of Items**	The number of items per page varied between 1 – 9. The full survey comprised a total of 41 items, although because of the adaptive nature of the questionnaire, not all respondents answered all items.
	**Number of screens (pages)**	The full survey was distributed over approximately 18 pages.
	**Completeness check**	Most survey items were mandatory (outstanding items were completed by 26/27 of respondents)<br>Most items, provided a non-response option such as "not applicable" or "rather not say", and selection of one response option was enforced.
	**Review step**	A Back button was available on each page, however respondents were unable to change their responses once submitted.
**Response Rates**	**Unique site visitor**	We asked respondents to identify the organization they were representing (information only available to the Principal Investigator). Decision-making regarding duplicates and inconsistencies in data provided by the respondents were discussed and unanimously decided in the multi-stakeholder working group.
	**View rate (Ratio of unique survey visitors/unique site visitors)**	Necessary detail for calculation was not recorded.
	**Participation rate (Ratio of unique visitors who agreed to participate/unique first survey page visitors)**	Necessary detail for calculation according to the proposed formula was not recorded. However, the survey had a participation rate of 64%, since 27/42 invited patient representatives submitted a response.

For peer review only

	**Completion rate (Ratio of users who finished the survey/users who agreed to participate)**	Necessary detail for calculation was not recorded.
**Preventing multiple entries**	**Cookies used**	Cookies were not used.
	**IP check**	IP addresses were not collected.
	**Log file analysis**	The study did not include a log file analysis.
	**Registration**	Not applicable.
**Analysis**	**Handling of incomplete questionnaires**	Only completed questionnaires were included in the final dataset.
	**Questionnaires submitted with an atypical timestamp**	No atypical timestamps were detected.
	**Statistical correction**	None required.

This checklist has been modified from Eysenbach G. Improving the quality of Web surveys: the Checklist for Reporting Results of Internet E-Surveys (CHERRIES). J Med Internet Res. 2004 Sep 29;6(3):e34 [erratum in J Med Internet Res. 2012; 14(1): e8.]. Article available at https://www.jmir.org/2004/3/e34/; erratum available https://www.jmir.org/2012/1/e8/. Copyright ©Gunther Eysenbach. Originally published in the Journal of Medical Internet Research, 29.9.2004 and 04.01.2012.

This is an open-access article distributed under the terms of the Creative Commons Attribution License (https://creativecommons.org/licenses/by/2.0/), which permits unrestricted use, distribution, and reproduction in any medium, provided the original work, first published in the Journal of Medical Internet Research, is properly cited.

For peer review only
